# Comparison of Temporal Transcriptomic Profiles from Immature Lungs of Two Rat Strains Reveals a Viral Response Signature Associated with Chronic Lung Dysfunction

**DOI:** 10.1371/journal.pone.0112997

**Published:** 2014-12-01

**Authors:** Elizabeth A. Hines, Renee J. Szakaly, Ning Leng, Anais T. Webster, Jamie M. Verheyden, Amber J. Lashua, Christina Kendziorski, Louis A. Rosenthal, James E. Gern, Ronald L. Sorkness, Xin Sun, Robert F. Lemanske

**Affiliations:** 1 Laboratory of Genetics, University of Wisconsin, Madison, Wisconsin, United States of America; 2 School of Pharmacy, University of Wisconsin, Madison, Wisconsin, United States of America; 3 Department of Medicine, University of Wisconsin, Madison, Wisconsin, United States of America; 4 Department of Statistics, University of Wisconsin, Madison, Wisconsin, United States of America; 5 Department of Pediatrics, University of Wisconsin, Madison, Wisconsin, United States of America; Children's Hospital Los Angeles, United States of America

## Abstract

Early life respiratory viral infections and atopic characteristics are significant risk factors for the development of childhood asthma. It is hypothesized that repeated respiratory viral infections might induce structural remodeling by interfering with the normal process of lung maturation; however, the specific molecular processes that underlie these pathological changes are not understood. To investigate the molecular basis for these changes, we used an established Sendai virus infection model in weanling rats to compare the post-infection transcriptomes of an atopic asthma susceptible strain, Brown Norway, and a non-atopic asthma resistant strain, Fischer 344. Specific to this weanling infection model and not described in adult infection models, Sendai virus in the susceptible, but not the resistant strain, results in morphological abnormalities in distal airways that persist into adulthood. Gene expression data from infected and control lungs across five time points indicated that specific features of the immune response following viral infection were heightened and prolonged in lungs from Brown Norway rats compared with Fischer 344 rats. These features included an increase in macrophage cell number and related gene expression, which then transitioned to an increase in mast cell number and related gene expression. In contrast, infected Fischer F344 lungs exhibited more efficient restoration of the airway epithelial morphology, with transient appearance of basal cell pods near distal airways. Together, these findings indicate that the pronounced macrophage and mast cell responses and abnormal re-epithelialization precede the structural defects that developed and persisted in Brown Norway, but not Fischer 344 lungs.

## Introduction

The inception of childhood asthma depends on interactions between environmental factors and development of the lung and immune system. For example, the relationship between infection with virus (e.g. rhinovirus or respiratory syncytial virus) and subsequent asthma onset is influenced by immune and lung factors [1]–[4]. The presence of allergic sensitization often precedes wheezing episodes and substantially increases the risk for subsequent asthma development following viral infection [3]–[5]. Alterations in innate immune responses to viral infection, particularly those related to interferon production, appear to contribute to abnormal host responses to these common respiratory pathogens and may be linked, at least in part, through IgE-dependent pathways [6]–[8]. Importantly, early life wheezing illnesses due to rhinovirus infections are associated with significant reductions in lung function in infancy and early childhood. The frequency and severity of wheezing respiratory tract illnesses in early life contribute to these reductions as well [9]–[12].

A number of animal models have been developed to further explore the relationships among viral respiratory infection, innate immune dysfunction, and the development of chronic lower respiratory tract diseases. For example, the effects of respiratory syncytial virus have been extensively studied in neonatal and adult mice and rats [13]. Both in mice [14] and in rats [15], [16], infection with Sendai virus causes pathologic changes in the lower airways that have many characteristics of human asthma. Interestingly, both the genetic strain [17]–[19] and the developmental stage of the rodents [14], [15], [20]–[22] at the time of infection contribute significantly to the risk of long term physiologic alterations such as airway obstruction [23] and hyperresponsiveness [20]. Innate immune responses, particularly those related to interferon production, may also have differential contributions to these long-term consequences [14], [24], [25].

Using a weanling rat model of virus-induced airway dysfunction, we have found that the development of a chronic asthma phenotype occurs in an “atopic” asthma susceptible strain (Brown Norway, BN) (Th2-like), but not in a non-atopic asthma non-susceptible strain (Fischer 344, F344) [24], [25]. We chose the Sendai virus, as it is a rodent parainfluenza type 1 virus that readily infects and replicates in rats, causing pulmonary pathology similar to respiratory syncytial virus in humans. Respiratory syncytial virus and rhinovirus replicate poorly in rodents, so that infection requires a massive inoculum, and the time course of replication is limited. Human parainfluenza viruses also can cause bronchiolitis in human, so that there is clinical analogy at least for the acute illness. For these reasons we elected to employ Sendai virus for the rat model, and we have more than 20 years of experience with this model [16], [25]. This model has many of the elements that parallel the development of the asthma in children: genetic susceptibility, immune dysfunction (atopy and innate immune response), induction of immune responses following viral infection, and developmental sensitivity to viral respiratory infection early in life. In particular, Sendai virus infection in the weanling BN rat results in structural changes of the lower airways that persist into adulthood, a phenotype that has not been described in studies of adult Sendai virus infection models [26]. These findings raise the hypothesis that strain-related differences in anti-viral immune responses and airway repair could lead to life-long changes in airway structure and physiology. Here, we carried out a comprehensive prospective microarray evaluation of lung tissues at multiple time points following viral infection in both the BN susceptible and F344 non-susceptible strains. Our results revealed genetic pathways that are associated with the histological and physiological changes in this rat model of asthma.

## Materials and Methods

### Ethics statement

The rats were housed and all experimental procedures were performed in an American Association for Accreditation of Laboratory Animal Care-accredited laboratory animal facility at the University of Wisconsin School of Medicine and Public Health. The study was approved by the University of Wisconsin School of Medicine and Public Health Animal Care and Use Committee (assurance number A3368-01) and conformed to the Guide for the Care and Use of Laboratory Animals.

### Animals

Male BN rats (SsN substrain; Harlan) and F344 rats (Charles River) were purchased and housed within HEPA-filtered isolation cubicles (Britz & Company, Wheatland, WY). All invasive procedures were performed under sodium pentobarbital anesthesia, and all efforts were made to minimize suffering.

### Virus inoculation and experimental design

Weanling (3–4 week old) BN and F344 rats were inoculated with aerosolized Sendai virus strain P3193 or vehicle (PBS) as previously described [25]. Briefly, weanling rats were exposed to an aerosol generated from stock fluid containing 2×10^8^ plaque-forming units (pfu) virus/ml, 2.5 ml of which was delivered into the chamber of a Glas-Col Aerosol Exposure Apparatus (Glas-Col, Terre Haute, IN) over 20 minutes. The apparatus can accommodate up to 50 weanling rats per inoculation batch. Noninfected controls were sham-inoculated in the same manner, using an aerosol of sterile vehicle (PBS). In previous inoculations using the same virus in this weanling rat model, it was shown that the viral titer peaks at 3–5 days post-inoculation, and was largely cleared by day 7, the earliest time point assayed here [25], [27]. In this study, to elucidate the molecular changes in the acute and chronic phases following infection, at 7, 10, 14, 21, and 35 days post-inoculation, rats were anesthetized, exsanguinated, and lungs were flash frozen in liquid nitrogen (n = 15 rats per group at each time point). Lung tissues for histology and RNA extraction came from separate, but identically controlled infection experiments.

### Histology

Lung fixation, processing, and embedding were performed as previously described [28]. Sagittal sections of the left lung were obtained and stained with hematoxylin and eosin.

### Immunofluorescent Staining

Immunofluorescent staining was carried out using standard protocols and citric acid antigen retrieval. The primary antibodies used were: mouse anti-CD68 ED1 at 1∶100 (Abcam), mouse anti-mast cell tryptase at 1∶500 (Abcam), mouse anti-P63 4A4 (Santa-Cruz Biotechnology), and rabbit anti-KRT5 (Covance). The secondary antibodies used were goat anti-mouse CY3 1∶200 and goat anti-rabbit FITC (Jackson Immunoresearch).

### RNA Extraction and Microarray Hybridization

Total lung RNA was isolated using RNeasy Mini Kit (Qiagen). Two micrograms of total RNA from each of the 15 rats in each group was pooled (in an effort to capture biological variation) [29] and submitted to the UW Gene Expression Center where the RNA was labeled and hybridized to one AFX Rat Genome 230 2.0 array per condition per time point (Affymetrix).

### Microarray Data Analysis

#### Statistical Methods

All analyses were carried out in R (R Development Core Team 2013).

#### Pre-processing and normalization

The Affymetrix probe level data was processed using Robust Multi-Array Analysis (RMA) to obtain normalized summary scores of expression for each probe set on each array [30]. All array data can be found in the NCBI-GEO database using the accession number GSE51267.

#### Identification of Differentially Expressed Genes

An empirical Bayes hierarchical modeling approach implemented in the R package *EB-HMM* was used to identify genes differentially expressed across multiple biological conditions over time. The approach has been previously described in detail [31]. *EB-HMM* extends EBarrays, an empirical Bayesian method for identifying differentially expressed genes at a single time point across two or more conditions [32], [33]. Specifically, EBarrays may be used to calculate gene-specific posterior probabilities of each possible expression pattern. For data in two conditions, there are two expression patterns: a gene may be differentially expressed (DE) or equivalently expressed (EE). For three conditions, there are five patterns: all three conditions are the same; all are different; or the first, second, or third conditions exhibit expression different from the other two. *EB-HMM* uses a hidden Markov model to accommodate dependence in expression patterns over time and like EBarrays, for every gene, provides a gene-specific distribution that quantifies the posterior probability of that gene being in each particular expression pattern. The posterior probabilities may be used to generate false discovery rate (FDR) controlled lists of genes. Here, a gene is assigned into a particular expression pattern if its posterior probability of being in that pattern exceeds 0.95. This threshold was chosen as it controls the FDR at 5%.

#### Principal component analysis (PCA)

PCA was performed using R function prcomp (R Development Core Team 2013). Prior to applying the PCA, the normalized gene expressions were rescaled to mean 0 and standard deviation 1 for each gene.

### Gene-set analysis based on gene ontology (GO) functional categories

The random-set scoring method was used to measure the enrichment of gene sets defined by GO functional categories [34]. The binary indicators of selection were built based on the genes identified in the previous differential expression analysis. The analysis was performed using R package *allez*. Gene-sets with Z-scores above 5 were considered enriched as suggested in the approach [34].

### Quantitative Real-Time PCR

A set of rats independent of those used for the microarray experiments was inoculated, harvested, and total RNA was prepared. RNA was reverse transcribed into cDNA using SuperScript III reverse transcriptase following the manufacturer's instructions (Invitrogen, Carlsbad, CA). Quantitative real-time PCR was performed using an ABI 7500 Real Time PCR System (Applied Biosystems, Foster City, CA). PrimeTime primer and probe sets for rat *Il-1b*, *A2m*, *C3*, *Cfi*, *Tgfb3*, and *Gp2* were designed and purchased from Integrated DNA Technologies (IDT, Coralville, IA). Primers and probes were: *β-actin* F: GCCGTCTTCCCCTCCAT, *β-actin* R: AGGAGTCCTTCTGACCCATACC, *β-actin* probe: CCATCACACCCTGGTGCC: *Il-1β* F: CTGCAGCTGGAGAGTGTG, *Il-1β* R: CTCCACTTTGGTCTTGACTTCT, *Il-1β* probe: TCCCAAACAATACCCAAAGAAGAAGATGGA; *A2m* F: GAAGTGAGAGTGACAGTTCCAG, *A2m* R: GCTTCCCATAGGTGTATATTCCA, *A2m* probe: ACACGGACACATTCATCTCTTCCTCC; *C3* F: GTGTAGGCACGCTGGTG, *C3* R: CCTCGATCCTTAGTGTCGTTTG, *C3* probe: TGACCCAAGAGATAACCGACAGCC; *Cfi* F: AGTTTGGCAGAGTGTACCTTTA, *Cfi* R: GACTTGTTGGGAAATCTGCATC, *Cfi* probe: TAGCACACTACACCTGCCAAGCC. All values were normalized to *β-actin*. Standard curves were generated by serial dilutions of cDNA from the lungs of a Sendai virus-inoculated rat and used to determine relative expression levels.

## Results

### Strain differences in histology following acute infection

BN rats, but not F344 rats, exhibit long-term airway obstruction following respiratory Sendai virus infection during the weanling period [15], [19], [26]. After the initial inoculation with virus, the two strains have been shown to develop similar peak lung viral titers and to clear the virus over a similar time course [27]. Consistent with previous studies using this model, rats of both strains displayed changes in breathing pattern and decreased activity by the 4^th^ to 5^th^ day (D4 to D5) after exposure to the aerosolized virus. Both exhibited reduced body weight and delayed growth during the acute illness, with similar differences relative to the respective non-infected controls for the two strains (P>0.9 for strain-by-inoculation interaction), and mean body weight at D7 being 61% of the sham-inoculated controls (P<0.0001). To understand the morphological origins of BN airway obstruction, histological analyses were carried out on lung sections from infected and control rats of each strain (BN with virus, BN-virus; F344 with virus, F344-virus; BN with saline, BN-sal; F344 with saline, F344-sal) and harvested at 7, 10, 14, 21, and 35 days post-infection (D7, D10, D14, D21, and D35).

**Figure 1:**
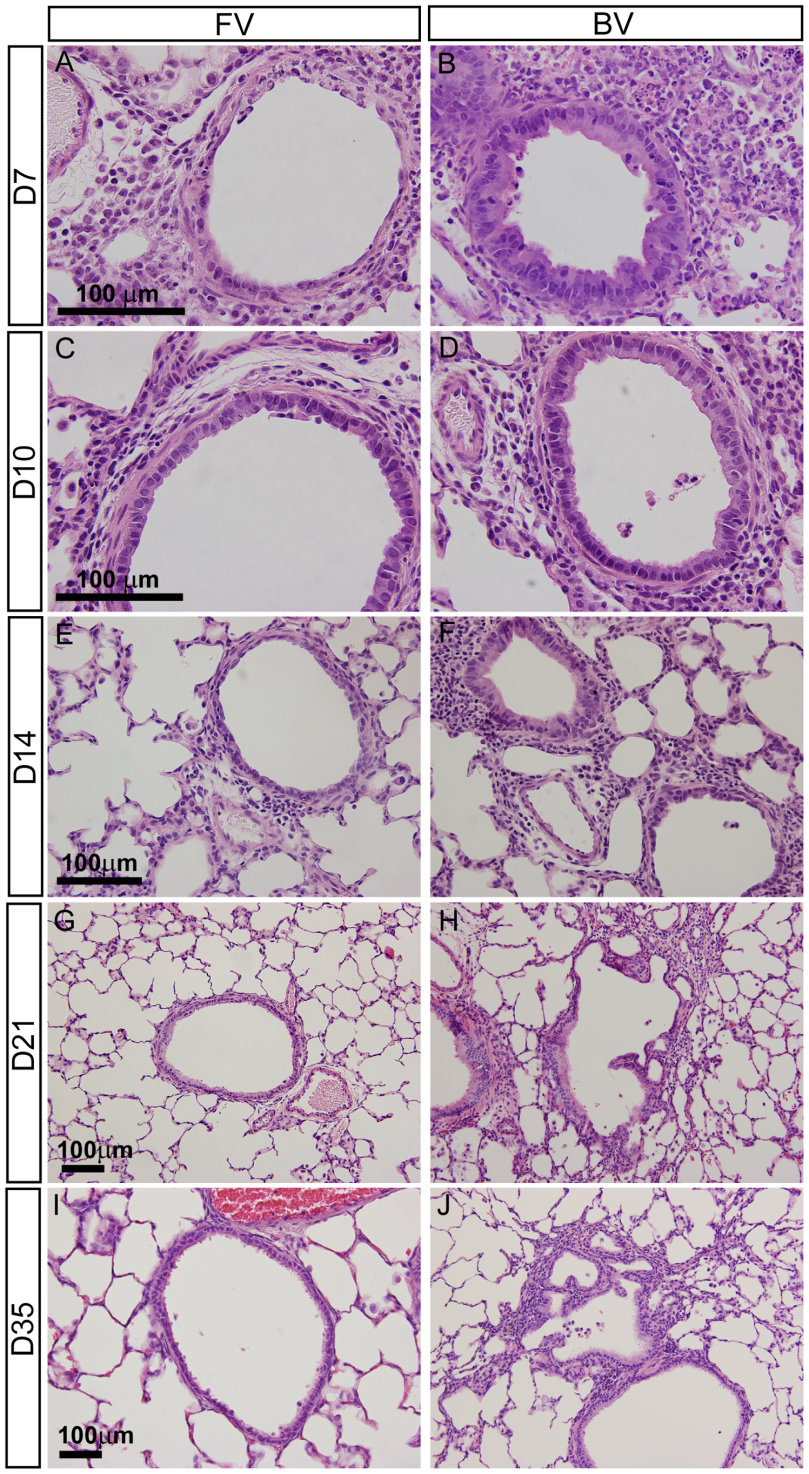
Lung sections comparing airway morphology changes in F344-virus and BN-virus lungs at representative time points. (A-J) H&E stained sections of F344-virus (FV) and BN-virus (BV) lungs at D7 (A-B), D10 (C-D), D14 (E-F), D21 (G-H), and D35 (I-J). Shown scale bars in micrometers (µm).

Histological analyses of infected and control tissue revealed clear temporal and strain-related differences in the appearance and resolution of specific histological features. D7 was characterized by a significant inflammatory response in both strains. BN-virus and F344-virus lungs exhibited terminal airway inflammation, necrosis, macrophage and neutrophil alveolar exudates, and perivascular edema (Figure 1A,B and Figure S1A-D). Focusing on the terminal airways, the epithelium of F344-virus airways was composed of cells with abnormal squamous morphology (Figure 1A). In contrast, BN-virus airways contained abnormally swollen epithelial cells (Figure 1B). By D10, the F344-virus airways were mostly restored to normal cuboidal morphology, while slightly swollen epithelium persisted in the BN-virus airways (Figure 1C,D and Figure S1E,F). F344-virus lungs, but not BN-virus lungs, had largely cleared neutrophils from their alveoli (data not shown). At D14, some BN-virus airways still contained taller epithelial cells and more inflammatory infiltrates than F344-virus airways (Figure 1E,F and Figure S1G,H). Starting at D21, F344-virus lungs largely resembled uninfected F344-sal lungs (Figure 1G,I and Figure S1I,K). In contrast, BN-virus lungs continued to exhibit lymphoid inflammation. In the terminal airways, the walls of a portion of these airways were composed of convoluted epithelium that formed sub-compartments that were either open or occluded from the main lumen (Figure 1H and Figure S1 J). These structures persisted in BN-virus lungs at D35, and are a unique feature of this strain following virus infection (Figure 1J and Figure S1L) [16]. Overall, compared to BN-virus lungs, F344-virus lungs exhibited a faster reduction of terminal airway inflammation, and a more complete restoration of normal epithelium.

### Gene ontology analysis of genes differentially expressed between BN-virus and F344-virus lungs

To elucidate the mechanisms of these post-infection structural differences, microarray analyses were performed using RNA collected from BN-virus, F344-virus, BN-sal, and F344-sal lungs at all five time points (n = 15 samples/experimental group). *EB-HMM* analysis identified genes that were differentially expressed between BN-virus and F344-virus (false discovery rate [FDR] of 5%). Principal component analysis (PCA) of the data show that the transcriptomes of BN-virus lungs at D7 and D10, and F344-virus lungs at D7 and D10 are most different from the transcriptomes of lungs at other conditions and time points (Figure S2). After D10, the transcriptomes of both strains start to return towards their sham-inoculated baseline, with the F344-virus lungs returning at a slightly faster timeline than BN-virus lungs.

Gene ontology (GO) analysis was then performed on differentially expressed genes using *allez*. The number of differentially expressed genes between BN-virus and F344-virus increased from 200 at D7 to 408 at D35, while the number of significant GO terms decreased from 137 at D7 to 6 at D35 (Table 1 and Table S1). These opposing changes of numbers suggest the early differentially expressed genes impact similar biological processes compared to the larger degree of functional heterogeneity performed by later differentially expressed genes.

**Table 1:** Total number of differentially expressed genes and significant GO terms at all five time points.

Days post-inoculation	D7	D10	D14	D21	D35
**# of genes differentially expressed between BN-virus and F344-virus**	200	209	520	470	408
**# of GO terms from differentially expressed genes**	137	59	27	21	6
**Range of GO term Z-scores**	10.317–5.006	10.547–5.112	7.218–5.153	7.352–5.206	5.944–5.031

**Figure 2:**
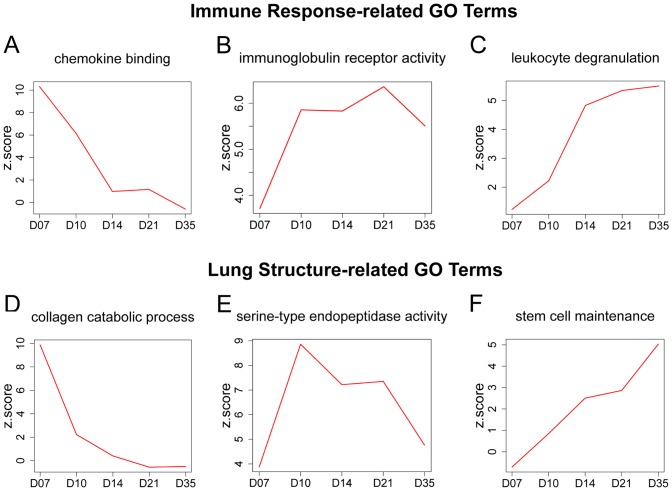
Gene ontology (GO) analysis of genes differentially expressed between BN-virus and F344-virus. (A-F) Z-score time course plots are shown for key GO terms related to immune response (A-C) or lung structure (D-F). Within each GO term plot, a higher Z-score indicates that more genes in that term group are differentially expressed between BN-virus and F344-virus.

The GO terms fell into two major categories: immune response-related genes and lung structure-related genes (Figure 2 and Table S2). We plotted the dynamic changes of Z-scores as a measure of how expression of genes in each GO term varies between BN-virus and F344-virus at comparable time points. Many immune response-related GO terms, such as “Chemokine Binding”, were enriched at early but not later time points (Figure 2A). The GO term “Immunoglobulin Receptor Activity” was representative of the types of terms enriched during the middle time points (D10-D21) (Figure 2B), while terms such as “Leukocyte Degranulation” became enriched during the later time points (D14-D35) (Figure 2C). The dynamics of these GO terms suggest a temporal progression of the strain-related differences from genes that function in recruitment of immune infiltrates, to activation of immune cell types, to local immune cell action.

For lung structure-related GO terms, “Collagen Catabolic Process” was most enriched at D7 and by D10 it was no longer significant (Figure 2D). During the middle time points, many terms related to peptidase activity were enriched similar to “Serine-type Endopeptidase Activity” (Figure 2E). By D35, there were few enriched GO terms; however, the “Stem Cell Maintenance” term exhibited significant enrichment during this time (Figure 2F).

### Patterns of strain differences in gene expression at baseline and following infection

**Figure 3:**
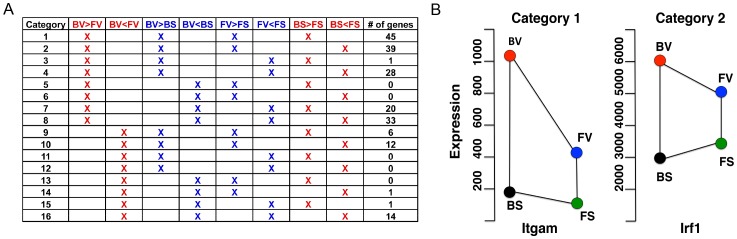
Analysis of inter-strain and intra-strain gene expression differences for D7 differentially expressed genes. (A) The 200 genes differentially expressed between BN-virus and F344-virus at D7 were sorted into the sixteen possible categories. Red columns indicate inter-strain comparisons: BN-virus (BV) vs. F344-virus (FV), BN-sal (BS) vs. F344-sal (FS). Blue columns indicate intra-stain comparisons: BN-virus (BV) vs. BN-sal (BS) and F344-virus (FV) vs. F344-sal (FS). The number of genes that fit the criteria in each row is listed on the right of each row. (B) As examples, strain differences in gene expression levels are diagramed for one selected gene for each of the two groups, Category 1 and Category 2, which contained the most genes. The x-axis represents no value other than to allow separate presentation of the two strains. The y-axis represents transcript level.

Strain-related differences in virus-induced gene expression could arise from differences in baseline expression or from the magnitude of induction. To determine the relative contribution of each mechanism, directional differences (increase vs. decrease) were identified through inter-strain (BN-virus vs. F344-virus, BN-sal vs. F344-sal) and intra-strain (BN-virus vs. BN-sal and F344-virus vs. F344-sal) comparisons. We grouped genes into sixteen categories based on possible inter- and intra- strain expression differences (Figure 3A). Here we present D7 differentially expressed genes as an example of findings from this analysis.

Of the 200 genes that were differentially expressed between BN-virus and F344-virus at D7 (5% FDR in *EB-HMM*), 172 genes were expressed at a higher level in BN-virus compared to F344-virus. It is interesting that when all 200 genes were subdivided into the sixteen categories, some categories contained no genes (Figure 3A and Table S3). On the other end of the spectrum, the two categories containing the most genes (Category 1, 45 genes; and Category 2, 39 genes) were patterns in which viral infection led to an increase of gene expression in both strains, but to a higher final level in BN rats (Figure 3B). The difference between Category 1 and Category 2 was the direction of baseline differences (Category 1: BN-sal>F344-sal, and Category 2: BN-sal<F344-sal). For genes in Category 2, they are expressed at a higher level in BN-virus compared to F344-virus, even though the baseline BN-sal was lower than F344-sal. This indicates that there is a larger magnitude of change following virus infection in BN (comparing BN-virus and BN-sal) than in F344 (comparing F344-virus and F344-sal). To determine if the same is true for Category 1 genes, the intra-strain expression increases was assessed (BN-virus to BN-sal compared with F344-virus to F344-sal) and showed that the BN increase in response to virus was at a minimum twice as large as the increase in F344. These data suggest that a larger response to virus in BN compared to F344 is a major pattern of gene expression differences as represented by Category 1 and 2 genes.

GO analysis was then performed for D7 on the categories that contained genes (Table S4). GO analysis on Category 1 and 2 genes demonstrated an enrichment of immune/inflammatory response genes. These data are consistent with our overall GO analysis data for the early time points.

### Visual categorization of temporal gene expression plots into inter/intra strain pattern groups

**Figure 4:**
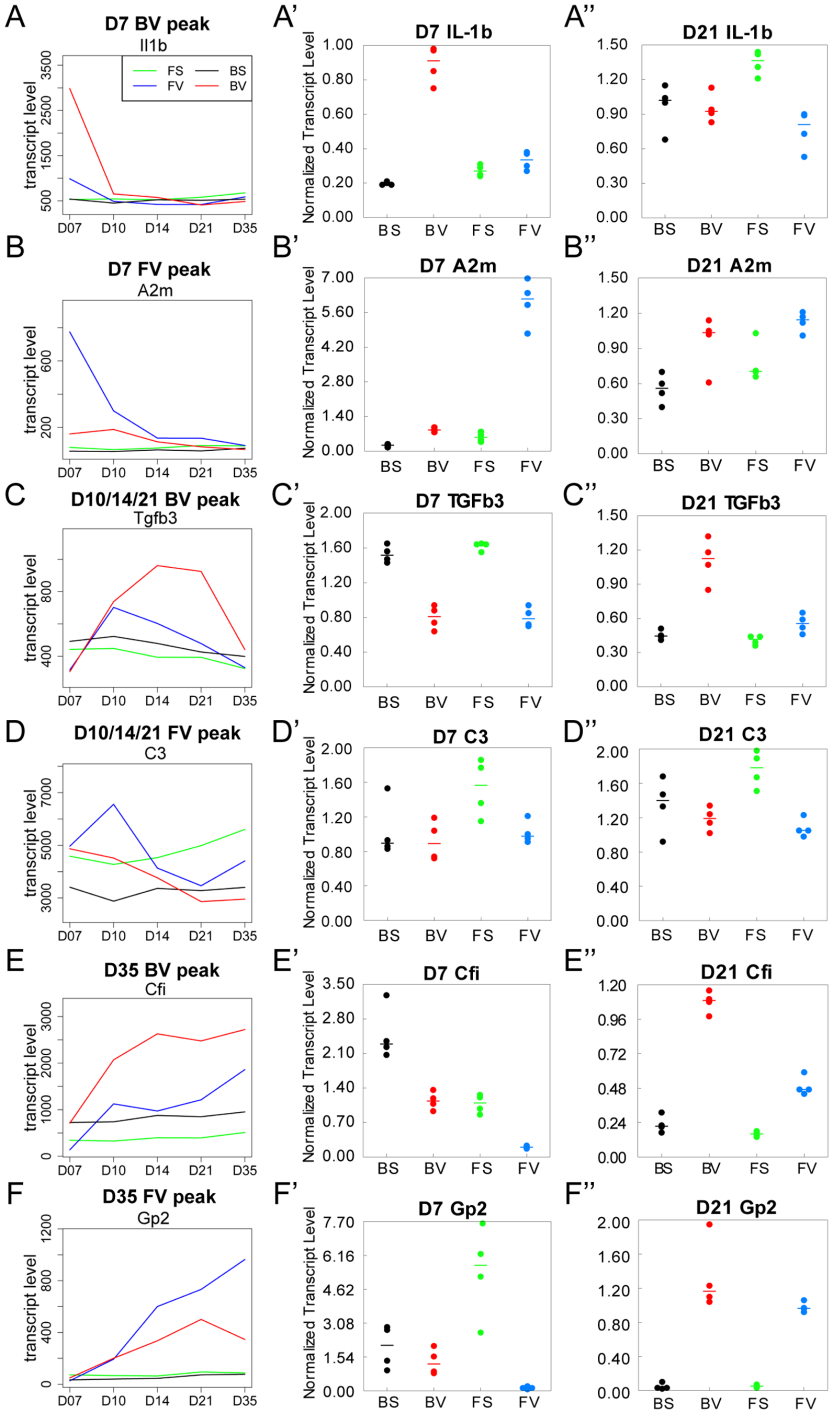
Representative time course plots for expression pattern groups. (A-F) Plots of individual genes that represent each pattern groups. Gene names are on top of each plot. The Y-axis indicates transcript level and the X-axis indicates the five time points. Inset in (A) indicates the color key for each line representing the expression level in F344-sal (FS) (green), BN-sal (BS) (black), F344-virus (FV) (blue), or BN-virus lungs (BV) (red). (A′-F′) qPCR verification of D7 gene expression levels. (A″-F″) qPCR verification of D21 gene expression levels. Dots in A′-F″ indicate values from four independent lung samples (biological replicates) and the line indicates the median value.

A temporal analysis was conducted on all differentially expressed genes being changed in at least one time point (5% FDR in *EB-HMM*). This allowed us to visualize the dynamic gene expression changes related to infection and rat strain over time. Three overall temporal expression patterns emerged and the genes were manually sorted into these groups: an early D7 peak, a middle D10/14/21 peak, and a late D35 peak (Figure 4 and Table S5).

The D7 peak group contained more genes than the other groups and was subdivided into two: the BN-virus peak subgroup and the F344-virus peak subgroup. Within the BN-virus D7 peak subgroup, there was an overrepresentation of genes in the cytokine pathway including 10 interleukin/interferon family members (*Il1b, Il12a, Il6, Il10, Il1r2, Il1rn, Il7r* and *Il8rb, Ifna1, Ifnb1, Ifng*) (Figure 4A, and Figure S3); 10 CCL type chemokines/receptors (*Ccl2,3,4,7,19,20*; *Ccr1,2,5*; *Ccrl2*) (Figure S4); 6 Stat family members (*Stat1,2,3,5a,5b,6*) (Figure S5); and 5 Irf family members (*Irf1,5,7,8,9*) (Figure S6). Similarly, there were 5 genes (*Areg, Egfr, Elf1, Elf5, Eps8*) associated with epidermal growth factor (EGF)/receptor tyrosine kinase (RTK) signaling, which has been implicated in virus-induced airway inflammation (Figure S7) [35]. Macrophage recruitment and activation genes were also enriched in this category. The similar dynamics of these genes further demonstrate that BN lungs experienced a heightened acute inflammatory response to viral infection compared with F344 lungs.

Within the F344-virus D7 peak subgroup, there was an enrichment of epithelial integrity and repair genes, including 4 keratins (*Krt4,5,14,19*), as well as *Mmp7*, and *Sprr1al* (and Figure S8). In addition, this F344-virus D7 subgroup also contained alpha2-macroglobulin (*A2m*), a known inhibitor of inflammatory molecules (Figure 4B) [36]. The D7 peak data suggest that pro-inflammatory genes are increased early in BN-virus lungs whereas epithelial repair and anti-inflammatory genes are increased early in F344-virus lungs.

The D10/14/21 peak group was also subdivided into two subgroups. In the BN-virus D10/D14/21 peak subgroup, there were a number of fibrosis related genes including *Tgfb1 and Tgfb3* (Figure 4C and Figure S9). Since TGFb acts in part to induce the expression of extracellular matrix (ECM) molecules, it is not surprising that ECM genes, *Cdh11, Col1a1/5a1/5a3, Ctgf*, and *Fn1* showed a similar pattern (Figure S9) [37]–[39]. This BN-virus peak group also contained the following genes previously implicated in asthma/allergy: high affinity immunoglobulin E receptors *Fcεr1a* and *Fcεr1b* (also known as *Ms4a2*), which are key genes in allergic responses [40]–[42]; *Tslp* which encodes a pro-inflammatory cytokine-like factor with polymorphisms linked to asthma susceptibility [43]; and *Cp*, which encodes an antioxidant and free radical scavenger produced by the airway under stressful conditions such as endotoxin administration (Figure S10) [44].

Distinct from the BN-virus D10/D13/21 subgroups, the F344-virus D10/D14/21 subgroup contained the genes *C3* and *C6* in the complement pathway, a system important for viral clearance and mast cell degranulation (Figure 4D and Figure S11) [45]–[47]. Also included in the F344-virus group were *Dmgdh* and *Bhmt2*, genes involved in the choline metabolism pathway, a source of key lipids involved in lung surfactant production and host defense against viral infection (Figure S11) [48]. Another gene in this F344-virus subgroup with a link to surfactants was *Ckap4*, which encodes a receptor for surfactant protein A (SPA) (Figure S11) [49]. The D10/14/21 peak subgroup data suggest a continued inflammation and emergence of fibrosis in BN-virus as the lung remodels, contrasted with activation of complement system and lipid metabolism in F344-virus as the lung repairs.

Compared to earlier peak groups, the D35 late peak group contained fewer genes. Included in the D35 BN-virus subgroup was *Cfi*, which encodes a serine protease that can degrade activated complement components such as aforementioned C3 (Figure 4E) [50]. Included in the D35 F344-virus subgroup was *Gp2*, which encodes a cell surface glycoprotein involved in mucosal response to bacteria (Figure 4F) [51]. Thus, a gradual increase of *Gp2* expression in F344-virus lungs may strengthen epithelium defense.

To validate the individual gene expression results from the array, we utilized quantitative real-time PCR (qPCR) to assess gene expression levels at D7 and D21 in an independent set of BN-virus, F344-virus, BN-sal, and F344-sal rats. We assessed expression of *Il1b, A2m, Tgfb3, C3, C6*, and *Gp2* in 4 rats from each category and found that the qPCR results correlated with the array results at the time points assessed, indicating that our array expression analysis is representative (Figure 4 A′-F′, A″-F″).

### BN-virus lungs exhibit increased macrophage gene expression and cell number

**Figure 5:**
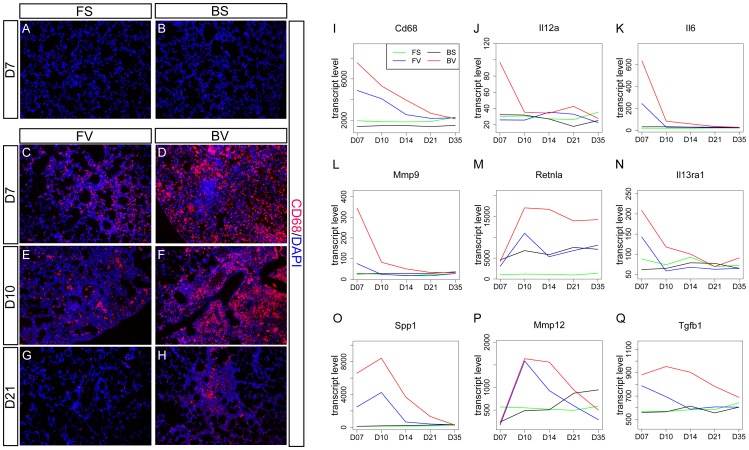
BN-virus lungs exhibit increased macrophage cell number and gene expression. (A-H) Lung sections stained using anti-CD68 antibody to label macrophages (red), and DAPI to label all nuclei (blue). CD68+ cells were rarely observed in either F344-sal (FS) (A) or BN-sal (BS) (B). A greater number of CD68+ cells were observed in BN-virus (BV) compared with F344-virus (FV) at D7 (C,D), D10 (E,F) and at D21 (G,H). Within each strain, there is a reduction in CD68+ cell number as time progresses after inoculation. (G-O) Gene expression plots of transcript level across five time points: the macrophage marker *Cd68* (I); an inductive signal for classically activated macrophages (CAMΦs) *Il12a* (J); CAMΦ effector genes *Il6* (K) and *Mmp9* (L); alternatively activated macrophage (AAMΦ) inductive signal receptor *Il13ra1* (M); AAMΦ marker *Retnla* (N); and AAMΦ effector genes *Spp1* (O), *Mmp12* (P), and *Tgfb1* (Q).

Key macrophage markers *Cd68*, *Cd14*, and *Cd163* were found in the D7 BN-virus peak group (Figure 5I and Figure S12) [52]–[54]. To determine if this heightened gene expression in BN-virus reflected an increased number of macrophages, an antibody against CD68 was used to label macrophages and their precursor monocytes. Consistent with gene expression data, at D7, more CD68+ cells were present in infected lungs compared to their respective controls. At both D7 and D10, more CD68+ cells were present in BN-virus compared with F344-virus (Figure 5A-F). By D21, macrophage/monocyte levels were decreased in both infected rat strains compared to earlier time points (Figure 5G,H). However, significant clusters of CD68+ cells were still observed near small airways in BN-virus lungs, but not in F344-virus lungs.

The inductive signals of classically activated macrophages (CAMΦs), *Il12a* and *Ifng*, exhibited greater expression in BN-virus than F344-virus at D7 (Figure 5J and Figure S3) [55], [56]. A similar pattern was observed with CAMΦ markers *Ifngr1/2, Irf1/5/7/8/9, Socs1, Tlr2, Tlr4*, and *Fcgr1a/2b/3a* (Figures S3 and S12). Consistent with this pattern, multiple effecter molecules produced by CAMΦs, including *Il6, Il1b, Ccl3/4*, *Tnfa*, and *Cxcl10*, also displayed increased expression in BN-virus lungs(Figure 4A, Figure 5K, Figure S4, Figure S13). BN-virus lungs had increased expression of multiple MMPs produced by CAMΦs, among other cell types, including *Mmp3/9/13* (Figure 5L and Figure S13). Similar to CAMΦs, expression of alternatively activated macrophage (AAMΦs) markers, *Retn1a, Mgl1*, and *Trem2*, was increased in BN-virus (Figure 5M and Figure S14) [57]. However, unlike CAMΦ makers that displayed peak expression at D7, the AAMΦ markers peaked in expression at D10. This delay suggests that CAMΦs may switch to AAMΦ in BN-virus lungs between D7 and D10, and/or there is a turnover of the macrophage cell population with the newly recruited macrophages taking on the AAMΦ phenotype [58]. Consistent with the former possibility, BN-virus lungs exhibited increased expression of genes involved in AAMØ induction within the D7-D10 time, including *Il13ra1* and *Il4ra* (Figure 5N and Figure S14). In addition, the expression of CAMΦ inhibitory signals, such as *Il1rn*, *Il10*, and *Ccl17*, was increased at D7 and D10 in BN-virus compared to F344-virus (Figure S3 and Figure S14). Furthermore, gene expression of AAMΦ effecter molecules such as *Spp1*, *MMP12/14*, and *TGFb1* continue to be expressed at D10 to D14 in BN-virus more than in F344-virus lungs (Figure 5 O-Q and Figure S14). Together with histological evidence, these data suggest that BN-virus lungs contain more macrophages than F344-virus lungs and that there may be a transition of CAMΦ to AAMΦ signatures in the BN-virus lungs.

### BN-virus lungs exhibit increased mast cell gene expression and cell number

**Figure 6:**
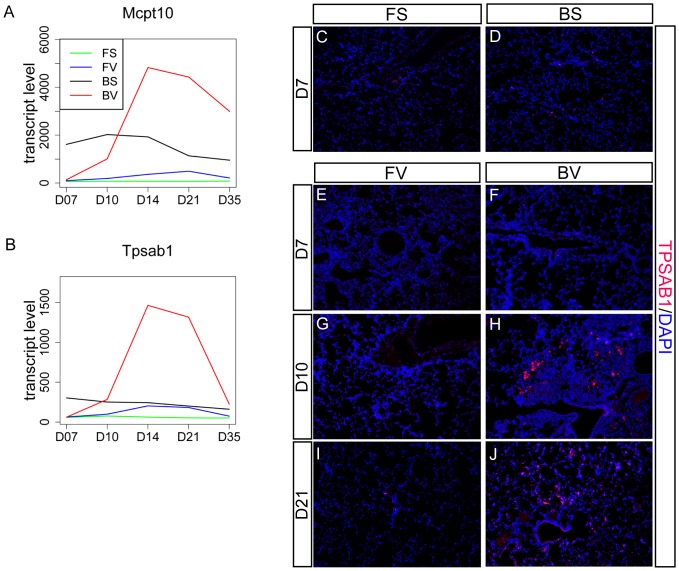
BN-virus lungs exhibit increased mast cell gene expression and cell number. (A,B) Expression plots of mast cell marker genes, protease *Mcpt10* (A) and tryptase *Tpsab1* (B). (C-J) Lung sections stained with anti-TPSAB1 antibody to label mast cells, and DAPI to label all nuclei (blue). For TPSAB1, no staining was observed in the F344-sal (FS) lung (C), infrequent staining was observed in BN-sal (BS) (D), D7, D10 or D21 F344-virus (FV) (E,G,I), D7 BN-virus (BV) lungs (F), and increased staining was observed in D10 and D21 BN-virus (BV) lungs (H,J).

Numerous mast cell-related genes were identified in the D10/D14/21 BN-virus peak group including *Mcpt1/3/8/9/10, Cma1, Cpa3, Tpsab1, and Tpsb2* (Figure 6A,B and Figure S15) [59], [60]. All of these genes showed greater expression in BN-sal than F344-sal at all five time points, indicating that mast cell genes have higher baseline expression in BN lungs. Viral infection produced little change of mast cell gene expression in F344 lungs. However, in BN lungs, viral infection caused mast cell gene expression to decrease below BN-sal levels at D7, after which gene expression in BN-virus lungs increased significantly, peaking at D14/21.

Consistent with the gene expression data, using an antibody against TPSAB1 to identify mast cells, low numbers were found in BN-sal and even fewer were found in F344-sal (Figure 6C,D). Additionally, while a low number of mast cells were found in F344-virus lungs across all five time points, an elevated number of mast cells were found in BN-virus lungs from D10 until D21 (Figure 6E-J). In BN-virus lungs, clusters of mast cells were observed preferentially at sites of fibrosis, suggesting a possible connection between mast cell produced catabolic enzymes and the structural changes observed in the vicinity.

### F344 lungs exhibit increased epithelial markers relative to BN lungs after viral infection

**Figure 7:**
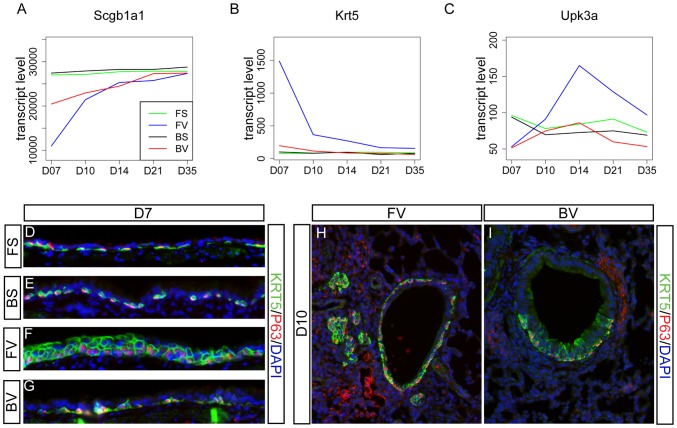
F344-virus exhibit efficient epithelial repair. (A-C) Gene plots of differentiated airway cell marker genes *Scgb1a1* for club cells (A); progenitor basal cells *Krt5* (B), and progenitor variant club cells *Upk3a* (C). (D-I) Longitudinal (D-G) or transverse (H-I) sections of the airway stained with basal cell markers anti-KRT5 (green) and P63 (red). Blue DAPI staining labels nuclei. Red staining in the mesenchyme in H and I is also observed in sham inoculated controls, and is not nuclear as expected for P63 expression. Thus, we speculate that it is non-specific background staining.

While the histological analysis shown in Figure 1 focused on terminal airways, gene expression changes that broadly represented the entire airways were also analyzed. Gene expression plots showed that differentiated epithelial cell markers, such as *Scgb1a1* for club cells and *Foxj1* for ciliated cells, were decreased in infected lungs compared to control lungs at early time points (Figure 7A and Figure S16) [61]. In contrast, expression of basal cell markers *Krt5* and *Krt14*, along with epithelial barrier-associated marker *Sprr1al*, was upregulated in F344-virus compared with BN-virus at D7 (Figure 7B and Figure S8) [62], [63]. Immunostaining with antibodies against markers KRT5 and P63, another key marker of basal cells [63], indicated that basal cells were indeed increased in F344-virus compared with BN-virus, F344-sal, and BN-sal at D7 and D10 (Figure 7D-I). In the pseudo-stratified epithelium of the most proximal airways, BN-sal, and F344-sal lungs displayed similar KRT5/P63 staining in cells closest to the basement membrane. BN-virus lungs had staining in the similar layer, albeit less uniform. Within F344-virus lungs however, the proximal airway epithelium was metaplastic and exhibited multiple layers of KRT5-positive epithelial cells. Additionally, KRT5/P63-positive cells extended into the distal airways of both BN-virus and F344-virus lungs, with greater coverage of the distal airways observed in F344-virus at D10 (Figure 7H-I).

Interestingly, KRT5/P63-positive pod-like structures were observed in F344-virus lungs at D10, but not in BN-virus, BN-sal, or F344-sal lungs at anytime. Epithelial pods have been previously observed in an H1N1 influenza model in mice, suggesting that a common repair mechanism may be activated following both Sendai virus infection in the F344-virus rat and H1N1 infection in the mouse [64]. The increase in basal cell number in infected tissue of the F344-virus lung was transient, as by D14 little differences were observed between viral infected and control lungs.

In addition to basal cell-associated repair, D14 F344-virus lungs exhibited increased expression of *Upk3a*, a marker of a recently identified lower airway epithelial progenitor population that can contribute to differentiated club and ciliated cells during repair (Figure 7C) [65]. These data suggest that the increased activity of more than one airway progenitor cell populations may be responsible for the more efficient repair of virus induced airway damage in the F344-virus lungs.

## Discussion

The weanling rat post-bronchiolitis model has proven valuable for providing mechanistic insights underlying the development of asthma in early life. Our current data extends previous work by interrogating the molecular pathways that contribute to complete resolution in non-susceptible (F344) lungs and the incomplete repair, aberrant remodeling, and persistent chronic airway physiologic dysfunction in susceptible (BN) lungs. Our data reveal two major mechanisms: first, differences in the magnitude and composition of the immune response, and second, differences in the resolution and/or repair of the tissue damage, which together may cause the persistent airway morphological and physiological defects observed in infected BN lungs.

It should be noted that the acute viral infection in this rat model is similar in time course and intensity for the two strains, with peak lung viral titers occurring at days 3–5 post inoculation, followed by a rapid clearance on days 6–8, with no virus detectable on plaque assay after day 8 [19], [25], [27]. Although the time points assayed in this study are all after virus clearance, the changes in breathing pattern and body weight observed here are consistent with our previous studies using this model. The time course of viral clearance in this rat model is similar to those reported for infant baboons and humans with acute respiratory syncytial virus infections [66].

Beginning from D7, the BN lungs mount an enhanced immune response compared to F344 lungs. Multiple interleukins, *CCL* type chemokines/receptors, *Stat*, and *Irf* family members exhibited enhanced BN-virus expression peaks at D7 compared to F344-virus lungs. The D7 BN-virus peak gene expression demonstrated a significant macrophage signature early, which then transitioned to a mast cell signature at later time points (D10-21).

Macrophages have been implicated in a number of respiratory illnesses. For example, in mice, macrophages are a major player in mediating neonatal carbon monoxide-induced lung injury leading to bronchopulmonary dysplasia [67], in influenza-induced immune signature and pathology [68], and in Sendai virus induced airway remodeling [69]. In our model, the macrophage signature can be further parsed based on temporal expression patterns. In BN-virus lungs, CAMΦ-associated genes were increased at D7 and AAMΦ-associated genes were increased at D10 in BN-virus compared to F344-virus lungs. Consistent with our current findings on transcript levels, previous studies demonstrated that TNFa and IL-13 proteins are increased in that BN-virus lungs compared to F344-virus lungs [19], [70]. Interestingly, while our results demonstrated increased expression of *Il12* and *Ifng* in BN-virus lungs compared to F344-virus lungs at D7, previous work using the same weanling model has shown the opposite protein level change at D1-3 post-infection [71]. This discrepancy could be due to a transient spike of IL12 and IFNg levels at D1-3 in F344-virus lungs, which may have returned to levels lower than those in BN-virus lungs by D7. Consistent with this possible time course, intraperitoneal injection of BN rats with recombinant IL-12 on the day of inoculation, but not two days after inoculation, inhibited the severity of virus-induced airway lesions in the BN rats [24], [71]. A number of effecter molecules made by CAMΦs and/or AAMΦs have been linked to downstream cellular responses including increased cell recruitment, mucus secretion, airway hyperresponsiveness and lung fibrosis. For example, CAMΦs release proinflammatory mediators such as TNFa that can lead to an excessive repair response and airway scarring [72], [73]. AAMΦs express MMP12 and SPP1 (osteopontin) which have been shown to be increased in asthma [74], [75].

Previous studies have shown that macrophages in BN-virus lungs exhibit higher TGFb1 expression relative to F344-virus lungs [76]. Our new findings are consistent with the prior results and demonstrate that the expression of several related genes is also increased. These include *Mmp9*, a key protease involved in cleaving TGFb pro-proteins, thereby activating its ability to signal [37]; a number of extracellular matrix (ECM) and adhesion molecules (*Fn, Col1a1, Col5a1, Col5a3, Fap* and *Cdh11*) [77]; and TGFb-induced fibrosis signals (*Ctgf* and *Il6*) [78], [79]. In addition, genes implicated in TGFb-induced smooth muscle cell contraction in asthmatic airways (*Il1b*, *Tnfa*, and *Ptgs1*) were also increased in BN-virus lungs [80]. There is therefore, a strong molecular signature of increased TGFb signaling that likely contributes to the prolonged fibrosis observed in BN lungs following viral infection.

The mast cell gene expression and immunostaining data presented here is consistent with prior results of greater mast cell numbers in BN-virus compared to F344-virus lungs [81]–[83]. This increase has been reported to arise from the proliferation of existing lung mast cells. Mast cells have also been shown to migrate in response to TGFb, and thereby localize to sites of inflammatory damage [84]. At D21, BN-virus lungs exhibited high expression of mast cell proteases, chymases, gelatinases, and tryptases, all of which are secreted molecules known to promote fibrosis [85]–[87]. Therefore, it is plausible that the local increase of mast cells in BN-virus lungs contributes to the development of the persistent fibrotic features of the small airways.

There is strong evidence that viral infection can cause epithelial barrier dysfunction [88], [89]. Conversely, an enhanced ability to repair the barrier could prevent viral-induced airways hyperresponsiveness. In our model, while expression of inflammatory response genes was increased in BN-virus lungs, F344-virus lungs exhibited increased expression of markers of epithelial repair as evidenced by transcript and cellular staining data. At D7 in the intra-pulmonary bronchi, stratified epithelium indicative of proliferation and healing was observed in F344-virus but not BN-virus. Consistent with an overall increase in basal cell marker expression, basal cells extend farther and cover distal airways more completely in F344-virus lungs compared to BN-virus lungs. This increased basal cell distribution is accompanied by increased *Mmp7*expression, which has been shown to stimulate epithelial cell migration in the repair process following denudation in both the mouse and human trachea [90], [91]. Additionally, pod-like structures comprised of basal cells were present in F344-virus lungs and not BN-virus lungs, consistent with the possibility that formation of these pods promotes airway repair. Combined with the increased expression of *Upk3a*, the lower airway progenitor marker, our results suggest that the increase in basal and UPK3A-positive progenitor cells may underlie the more effective and uniform repair in F344-virus airways compared to BN-virus airways.

A unique and persistent feature of the BN airway that may contribute to airflow obstruction and reduced lung function is the convolution and irregularity of the terminal airways [26]. These structures were first detected at D14. We speculate that these deformities may result from the persistence of immune cells, such as macrophages and mast cells, which secrete enzymes that remodel the extracellular matrix. The local enrichment of macrophages and mast cells in regions with irregular airways is consistent with this possibility. Alternatively or in addition, the deformities may be due to irregular and non-uniform repair of the airways. In support of this possibility, BN-virus lungs exhibited swollen and irregular small airways with partial coverage by p63-positive cells at D7 and D10, prior to the appearance of the aberrant airway morphology.

The virus-induced rat model of airway dysfunction reported herein provided the impetus for the design of the Childhood Origins of ASThma (COAST) study, an ongoing high risk prospective birth cohort study that has explored both environmental (respiratory tract infections) and genetic (immune response) factors that contribute to the inception of childhood asthma [92]. Similar to the rat model, early life immune dysregulation is associated with persistent wheezing in preschool children, and wheezing illnesses due to rhinovirus in the first three years of life are associated with persistent and chronic loss of airway function [9], and are very significant risk factors for the subsequent development of asthma by age six years [2], [93], [94]. Moreover, certain genetic loci are highly predictive in terms of asthma risk in those children who have experienced rhinovirus wheezing illnesses [95]. Genetic variations likely underlie the highly reproducible differences seen in the response of the two rat strains to the same Sendai virus infection. The accessibility of this rat model then poses it as an ideal system for the interrogation of genetic basis of the differential viral responses, using inter-strain mating, mapping and high density sequencing approaches.

Due to lack of samples, it has not been shown whether persistent lung or airway remodeling occur in human infants following rhinovirus or respiratory syncytial virus infections. However, both rhinovirus and respiratory syncytial virus have been shown to induce similar factors as observed in the rat model here. These include chemokines such as CXCL10 [96], [97], and factors that have been implicated in remodeling [98]–[100]. Thus, we expect that our newly generated microarray data from the rat model can be used to further interrogate the existence of similar molecular pathways in humans. These shared molecular mechanisms may contribute to airway structural changes and dysfunction following respiratory viral infection that occurs at a critical time of the co-development of the immune system and lung in both species.

## Supporting Information

Figure S1 **Histological lung sections comparing global morphology of FS, BS, FV, and BV at representative time points.** (A-L) Less magnified views of H&E stained lung sections of control F344-sal (FS) and BN-sal (BS) at D7 (A,B) and F344-virus (FV) and BN-virus (BV) at D7 (C,D), D10 (E,F), D14 (G,H), D21 (I,J) and D35 (K,L). Shown scale bars in micrometers (µm).
(TIF)

Figure S2 **Principle component analysis (PCA).** PCA was performed using transcriptome-wide gene expression profile. The x axis and the y axis show the first 2 principle components respectively. Four strains are shown in different colors.
(TIF)

Figure S3 **Additional D7 BN-virus peak gene plots.** Gene names indicated. F344-sal (FS) (green), BN-sal (BS) (black), F344-virus (FV) (blue), or BN-virus lungs (BV) (red).
(TIF)

Figure S4 **Additional D7 BN-virus peak gene plots.** Gene names indicated. F344-sal (FS) (green), BN-sal (BS) (black), F344-virus (FV) (blue), or BN-virus lungs (BV) (red).
(TIF)

TIF Figure S5 **Additional D7 BN-virus peak gene plots.** Gene names indicated. F344-sal (FS) (green), BN-sal (BS) (black), F344-virus (FV) (blue), or BN-virus lungs (BV) (red).
(TIF)

Figure S6 **Additional D7 BN-virus peak gene plots.** Gene names indicated. F344-sal (FS) (green), BN-sal (BS) (black), F344-virus (FV) (blue), or BN-virus lungs (BV) (red).
(TIF)

Figure S7 **D7 BN-virus peak gene plots.** Gene names indicated. F344-sal (FS) (green), BN-sal (BS) (black), F344-virus (FV) (blue), or BN-virus lungs (BV) (red).
(TIF)

Figure S8 **Additional D7 F344-virus peak gene plots.** Gene names indicated. F344-sal (FS) (green), BN-sal (BS) (black), F344-virus (FV) (blue), or BN-virus lungs (BV) (red).
(TIF)

Figure S9 **Additional D10/14/21 BN-virus peak gene plots.** Gene names indicated. F344-sal (FS) (green), BN-sal (BS) (black), F344-virus (FV) (blue), or BN-virus lungs (BV) (red).
(TIF)

Figure S10 **Additional D10/14/21 BN-virus peak gene plots.** Gene names indicated. F344-sal (FS) (green), BN-sal (BS) (black), F344-virus (FV) (blue), or BN-virus lungs (BV) (red).
(TIF)

Figure S11 **Additional D10/14/21 F344-virus peak gene plots.** Gene names indicated. F344-sal (FS) (green), BN-sal (BS) (black), F344-virus (FV) (blue), or BN-virus lungs (BV) (red).
(TIF)

Figure S12 **Additional classically activated macrophage marker gene plots.** Gene names indicated. F344-sal (FS) (green), BN-sal (BS) (black), F344-virus (FV) (blue), or BN-virus lungs (BV) (red).
(TIF)

Figure S13 **Additional classically activated macrophage effector molecule gene plots.** Gene names indicated. F344-sal (FS) (green), BN-sal (BS) (black), F344-virus (FV) (blue), or BN-virus lungs (BV) (red).
(TIF)

Figure S14 **Additional alternatively activated macrophage-related gene plots.** Gene names indicated. F344-sal (FS) (green), BN-sal (BS) (black), F344-virus (FV) (blue), or BN-virus lungs (BV) (red).
(TIF)

Figure S15 **Additional mast cell-related gene plots.** Gene names indicated. F344-sal (FS) (green), BN-sal (BS) (black), F344-virus (FV) (blue), or BN-virus lungs (BV) (red).
(TIF)

Figure S16 **Additional ciliated cell marker gene plot.** F344-sal (FS) (green), BN-sal (BS) (black), F344-virus (FV) (blue), or BN-virus lungs (BV) (red).
(TIF)

Table S1 **List of all differentially expressed genes at each time point.**
(XLSX)

Table S2 **List of all significant GO groups at each time point.**
(XLSX)

Table S3 **List of D7 differentially expressed genes by category.**
(XLSX)

Table S4 **GO terms for D7 Category 1 and Category 2 genes.**
(XLSX)

Table S5 **List of genes in the shape plot categories.**
(XLSX)
