# Supplementary figures and images for: Comparison of Temporal Transcriptomic Profiles from Immature Lungs of Two Rat Strains Reveals a Viral Response Signature Associated with Chronic Lung Dysfunction
Source: PLoS One. 2014 Dec 1;9(12):e112997. doi: 10.1371/journal.pone.0112997 (PMC4249857; doi:10.1371/journal.pone.0112997)

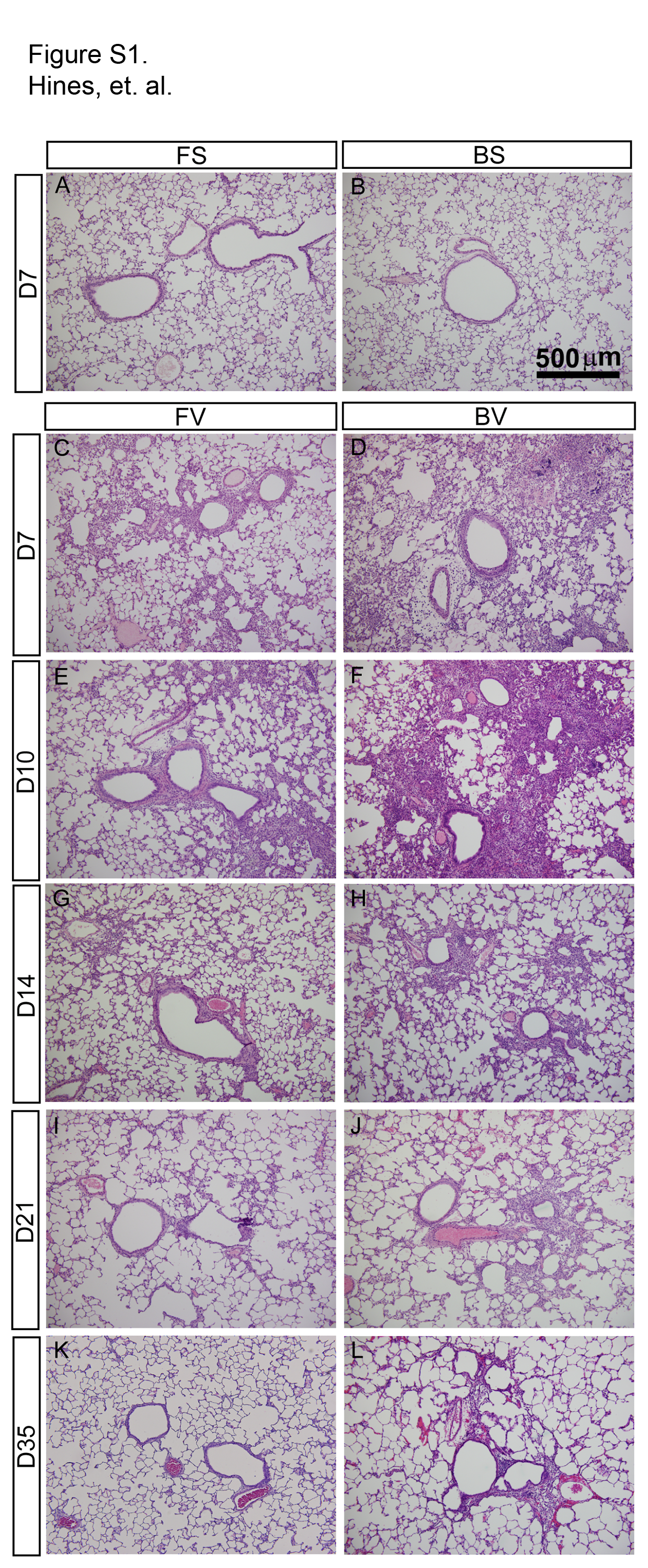

Supplement: Figure S1 — Histological lung sections comparing global morphology of FS, BS, FV, and BV at representative time points. (A-L) Less magnified views of H&E stained lung sections of control F344-sal (FS) and BN-sal (BS) at D7 (A,B) and F344-virus (FV) and BN-virus (BV) at D7 (C,D), D10 (E,F), D14 (G,H), D21 (I,J) and D35 (K,L). Shown scale bars in micrometers (µm). (TIF) [file pone.0112997.s001.tif]

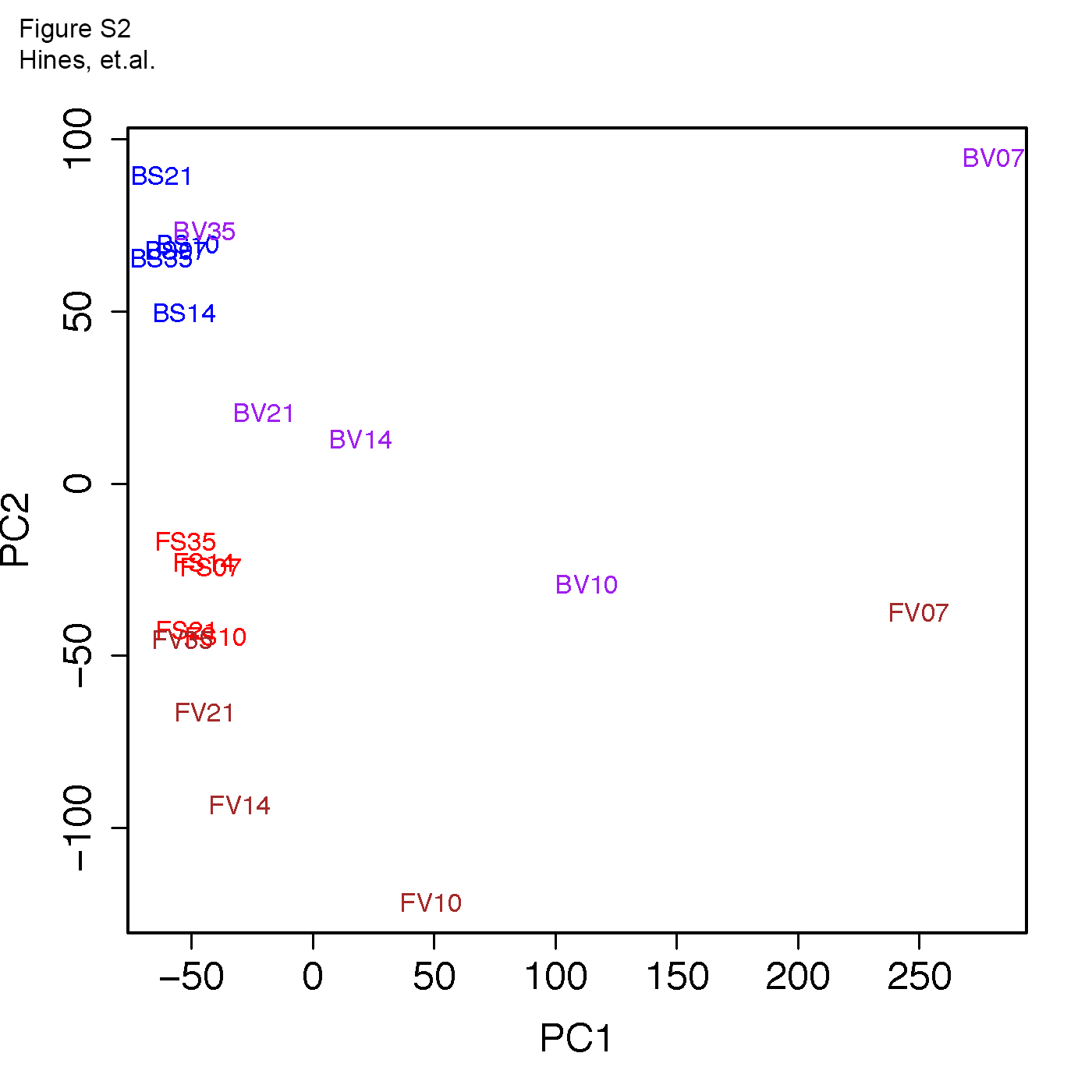

Supplement: Figure S2 — Principle component analysis (PCA). PCA was performed using transcriptome-wide gene expression profile. The x axis and the y axis show the first 2 principle components respectively. Four strains are shown in different colors. (TIF) [file pone.0112997.s002.tif]

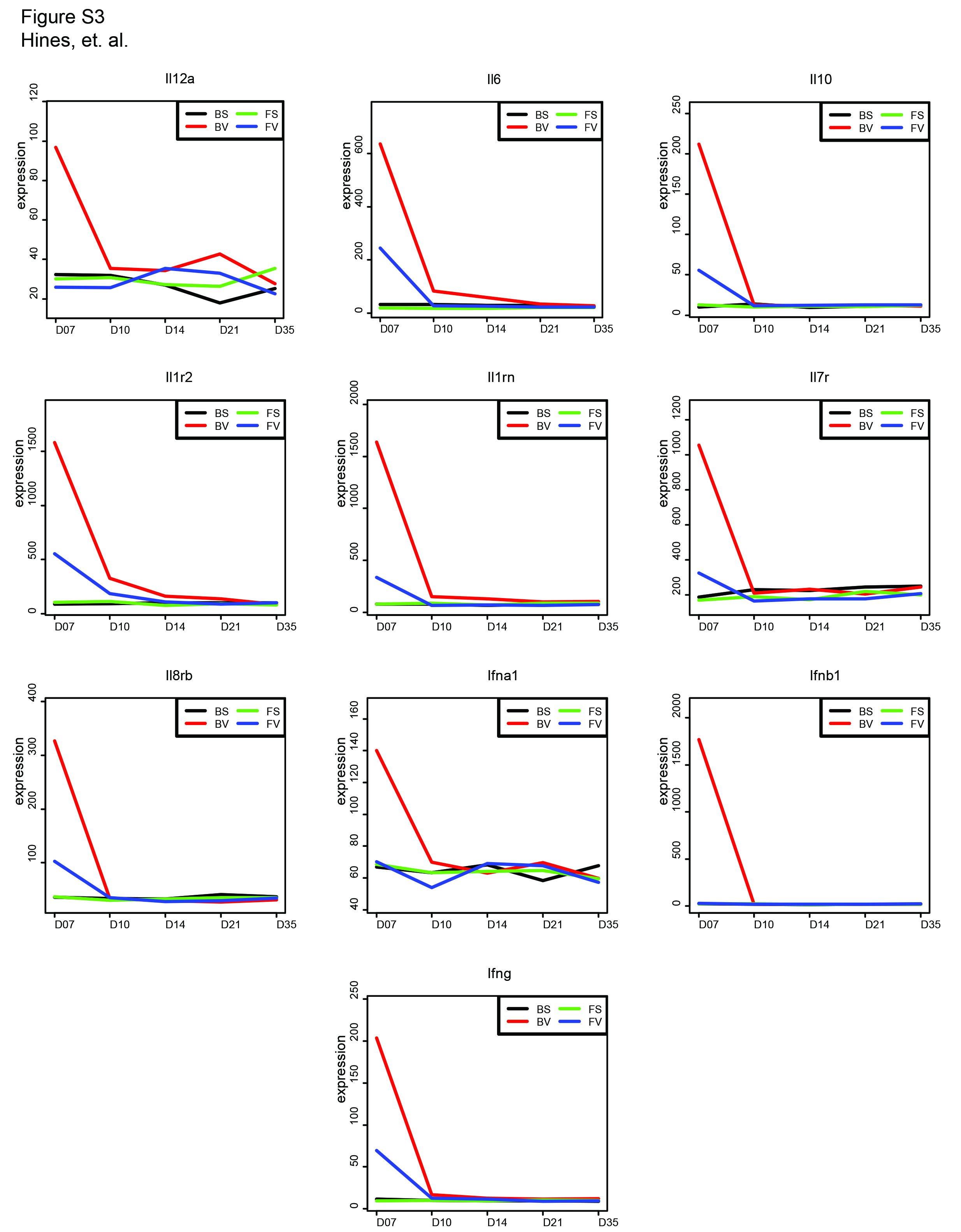

Supplement: Figure S3 — Additional D7 BN-virus peak gene plots. Gene names indicated. F344-sal (FS) (green), BN-sal (BS) (black), F344-virus (FV) (blue), or BN-virus lungs (BV) (red). (TIF) [file pone.0112997.s003.tif]

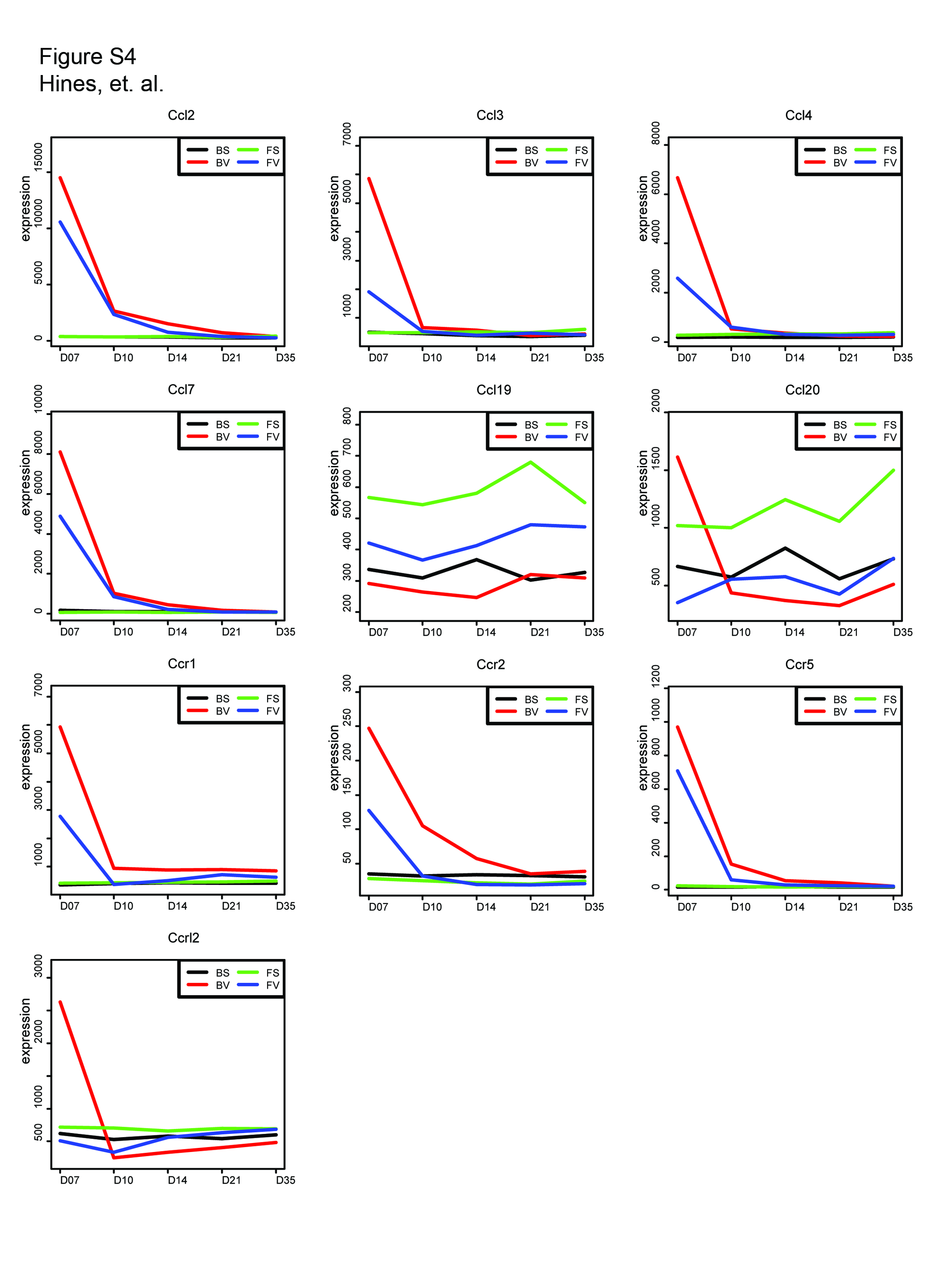

Supplement: Figure S4 — Additional D7 BN-virus peak gene plots. Gene names indicated. F344-sal (FS) (green), BN-sal (BS) (black), F344-virus (FV) (blue), or BN-virus lungs (BV) (red). (TIF) [file pone.0112997.s004.tif]

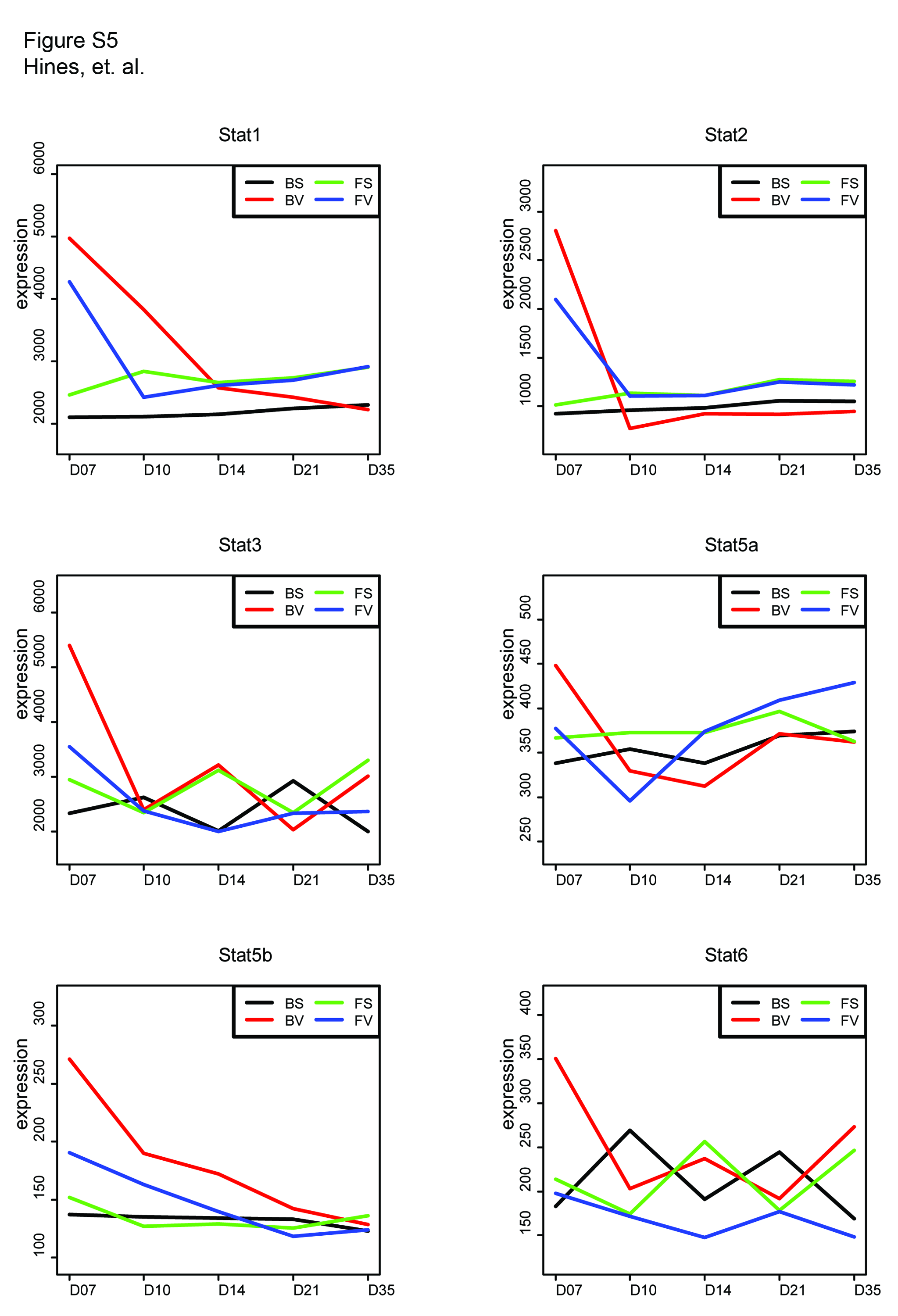

Supplement: TIF Figure S5 — Additional D7 BN-virus peak gene plots. Gene names indicated. F344-sal (FS) (green), BN-sal (BS) (black), F344-virus (FV) (blue), or BN-virus lungs (BV) (red). (TIF) [file pone.0112997.s005.tif]

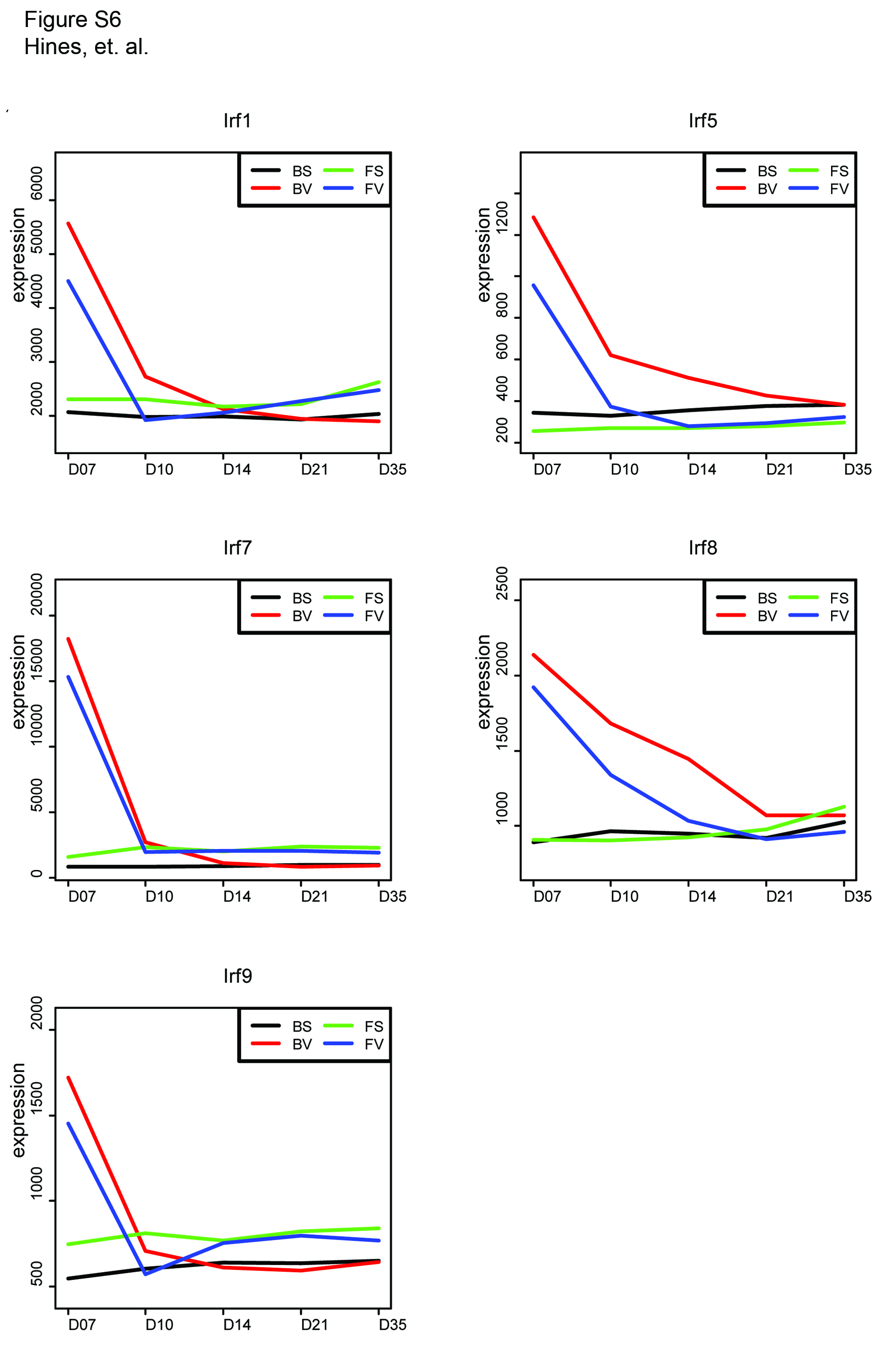

Supplement: Figure S6 — Additional D7 BN-virus peak gene plots. Gene names indicated. F344-sal (FS) (green), BN-sal (BS) (black), F344-virus (FV) (blue), or BN-virus lungs (BV) (red). (TIF) [file pone.0112997.s006.tif]

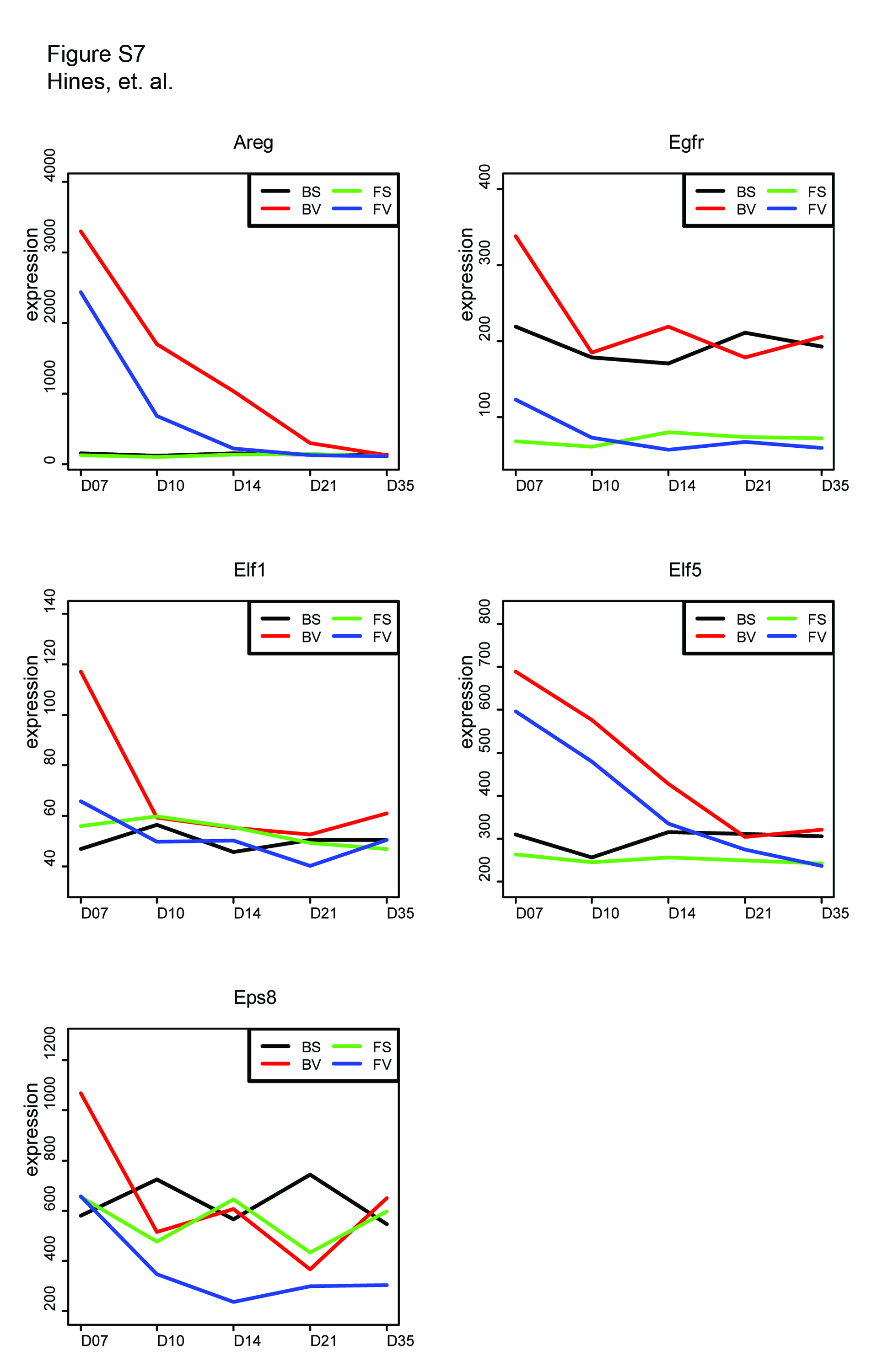

Supplement: Figure S7 — D7 BN-virus peak gene plots. Gene names indicated. F344-sal (FS) (green), BN-sal (BS) (black), F344-virus (FV) (blue), or BN-virus lungs (BV) (red). (TIF) [file pone.0112997.s007.tif]

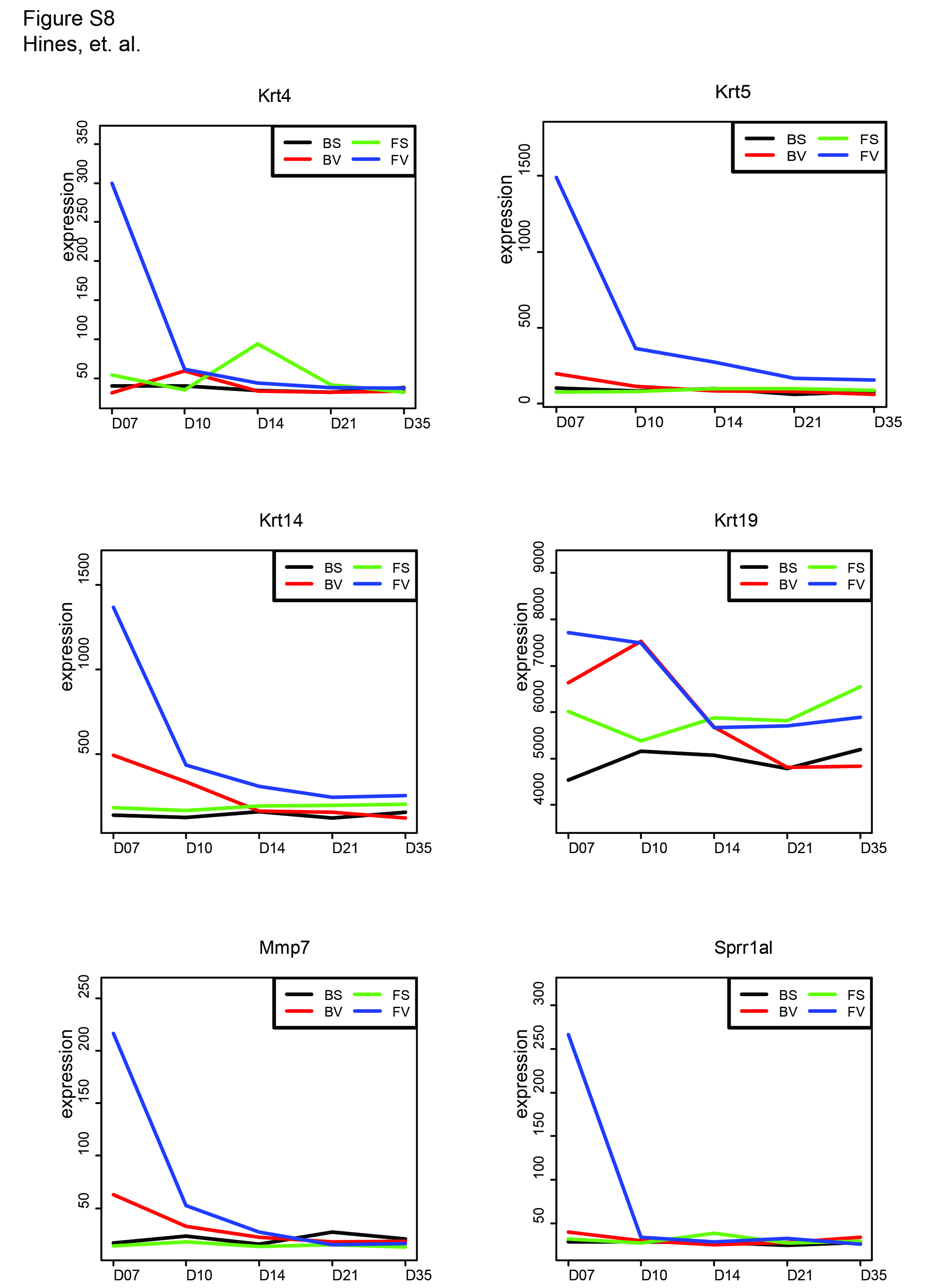

Supplement: Figure S8 — Additional D7 F344-virus peak gene plots. Gene names indicated. F344-sal (FS) (green), BN-sal (BS) (black), F344-virus (FV) (blue), or BN-virus lungs (BV) (red). (TIF) [file pone.0112997.s008.tif]

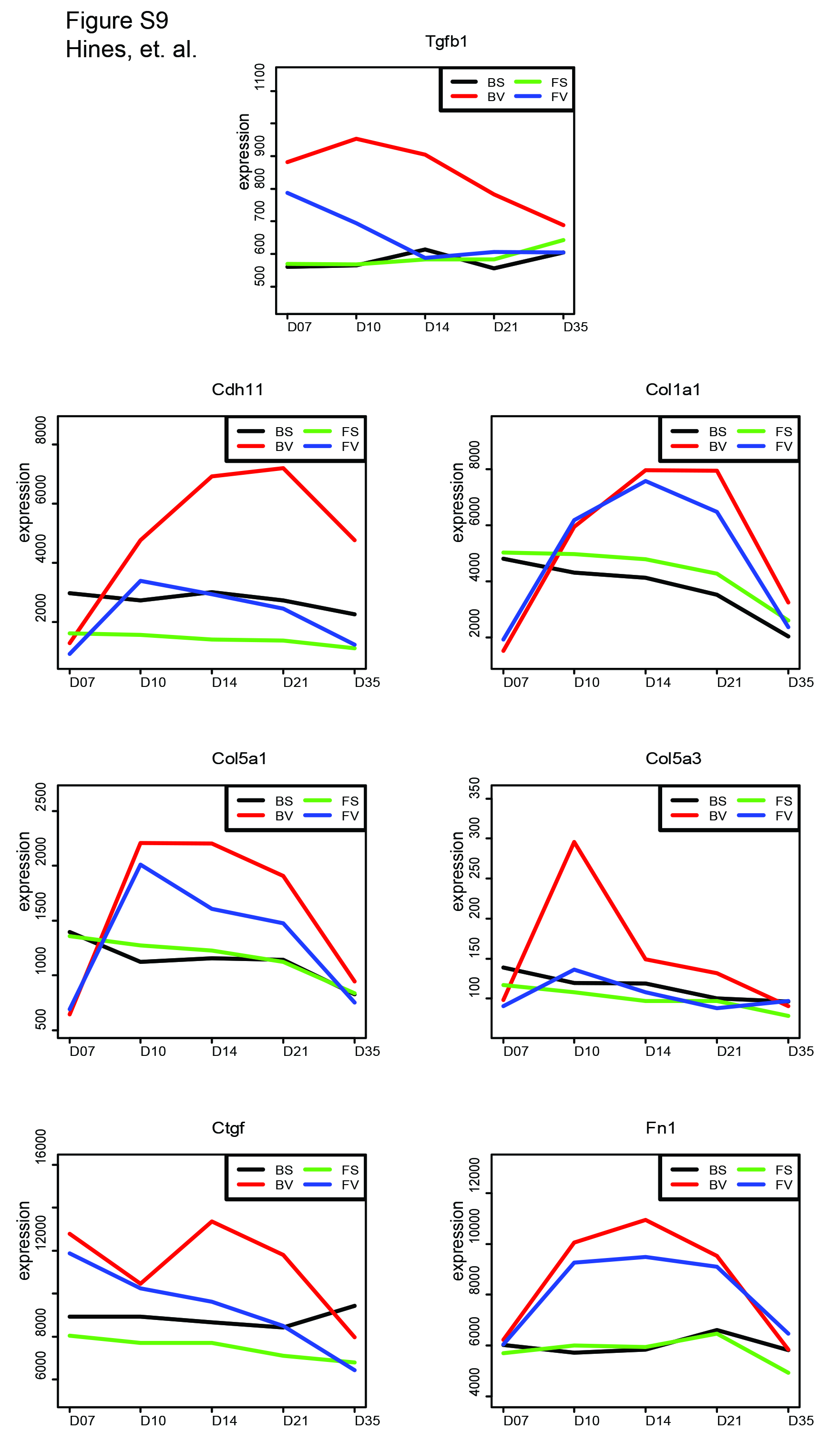

Supplement: Figure S9 — Additional D10/14/21 BN-virus peak gene plots. Gene names indicated. F344-sal (FS) (green), BN-sal (BS) (black), F344-virus (FV) (blue), or BN-virus lungs (BV) (red). (TIF) [file pone.0112997.s009.tif]

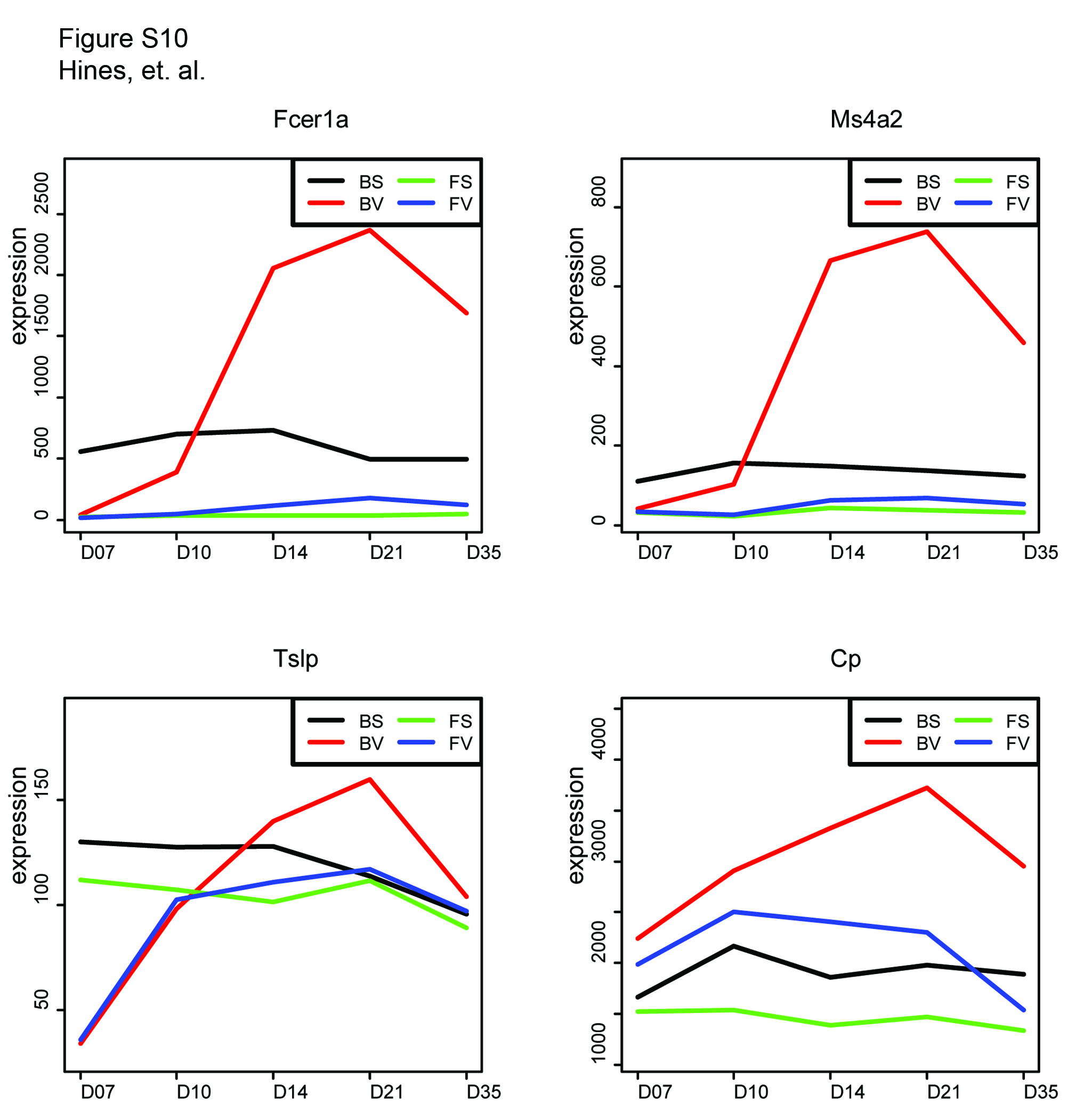

Supplement: Figure S10 — Additional D10/14/21 BN-virus peak gene plots. Gene names indicated. F344-sal (FS) (green), BN-sal (BS) (black), F344-virus (FV) (blue), or BN-virus lungs (BV) (red). (TIF) [file pone.0112997.s010.tif]

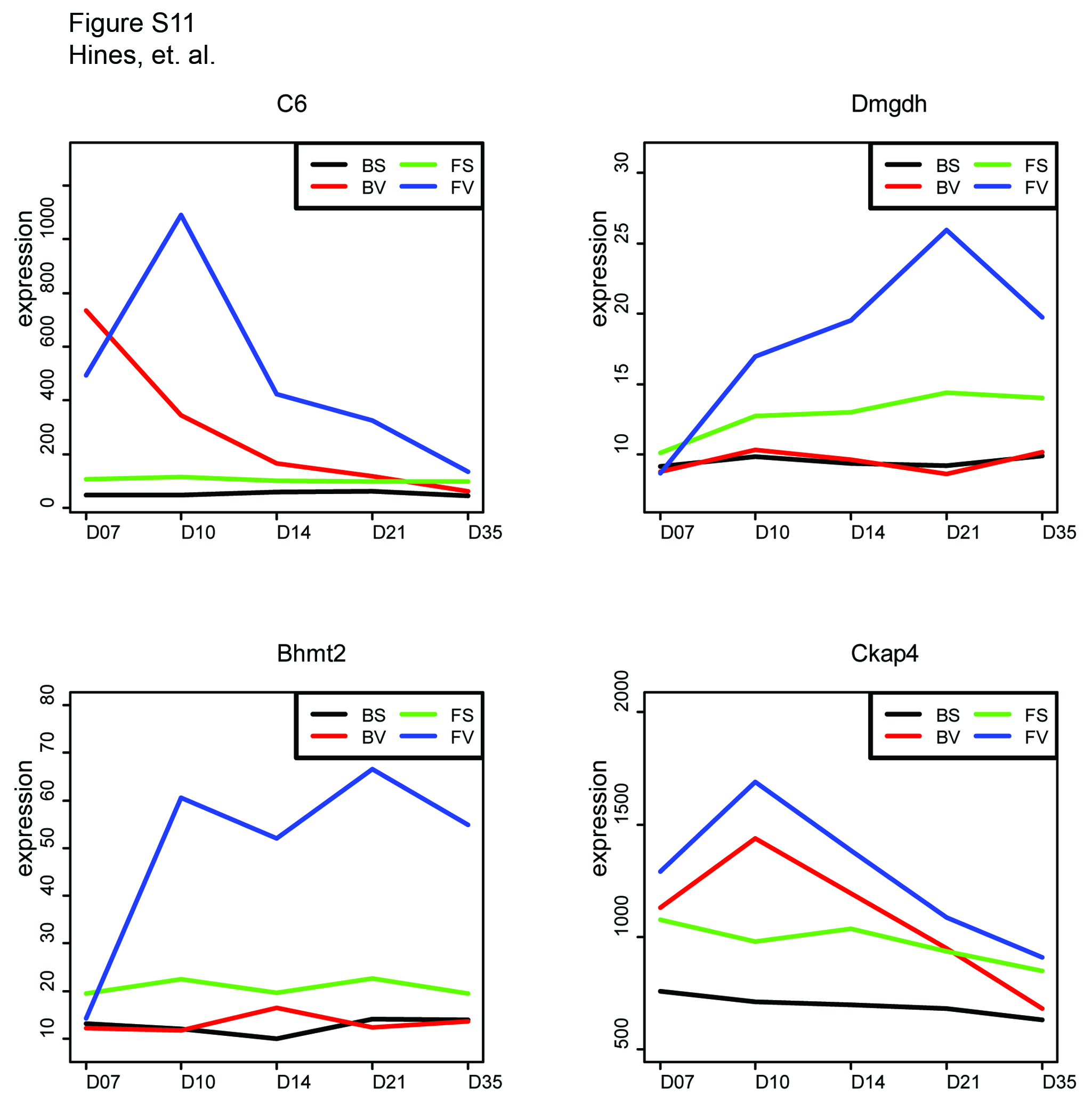

Supplement: Figure S11 — Additional D10/14/21 F344-virus peak gene plots. Gene names indicated. F344-sal (FS) (green), BN-sal (BS) (black), F344-virus (FV) (blue), or BN-virus lungs (BV) (red). (TIF) [file pone.0112997.s011.tif]

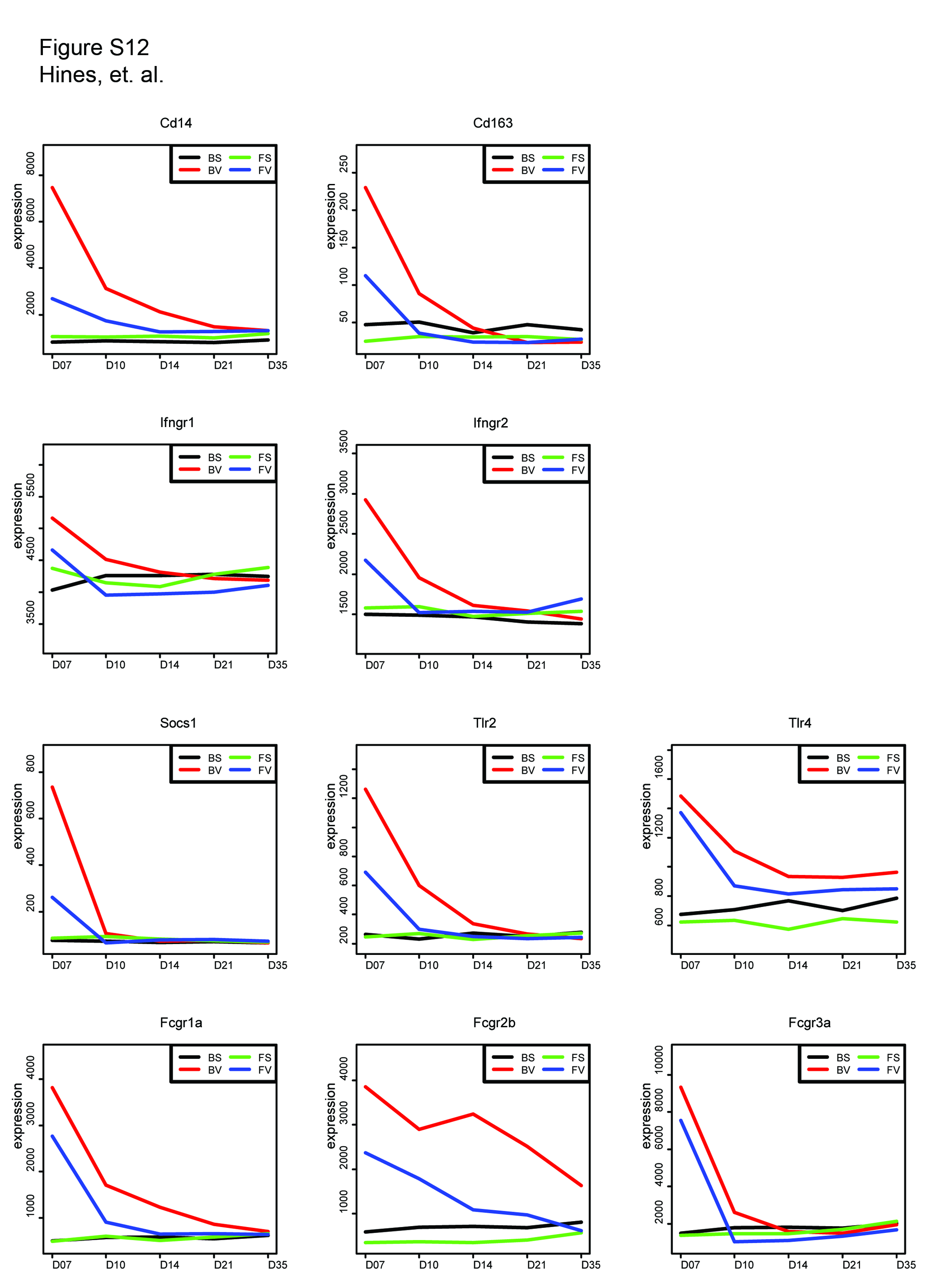

Supplement: Figure S12 — Additional classically activated macrophage marker gene plots. Gene names indicated. F344-sal (FS) (green), BN-sal (BS) (black), F344-virus (FV) (blue), or BN-virus lungs (BV) (red). (TIF) [file pone.0112997.s012.tif]

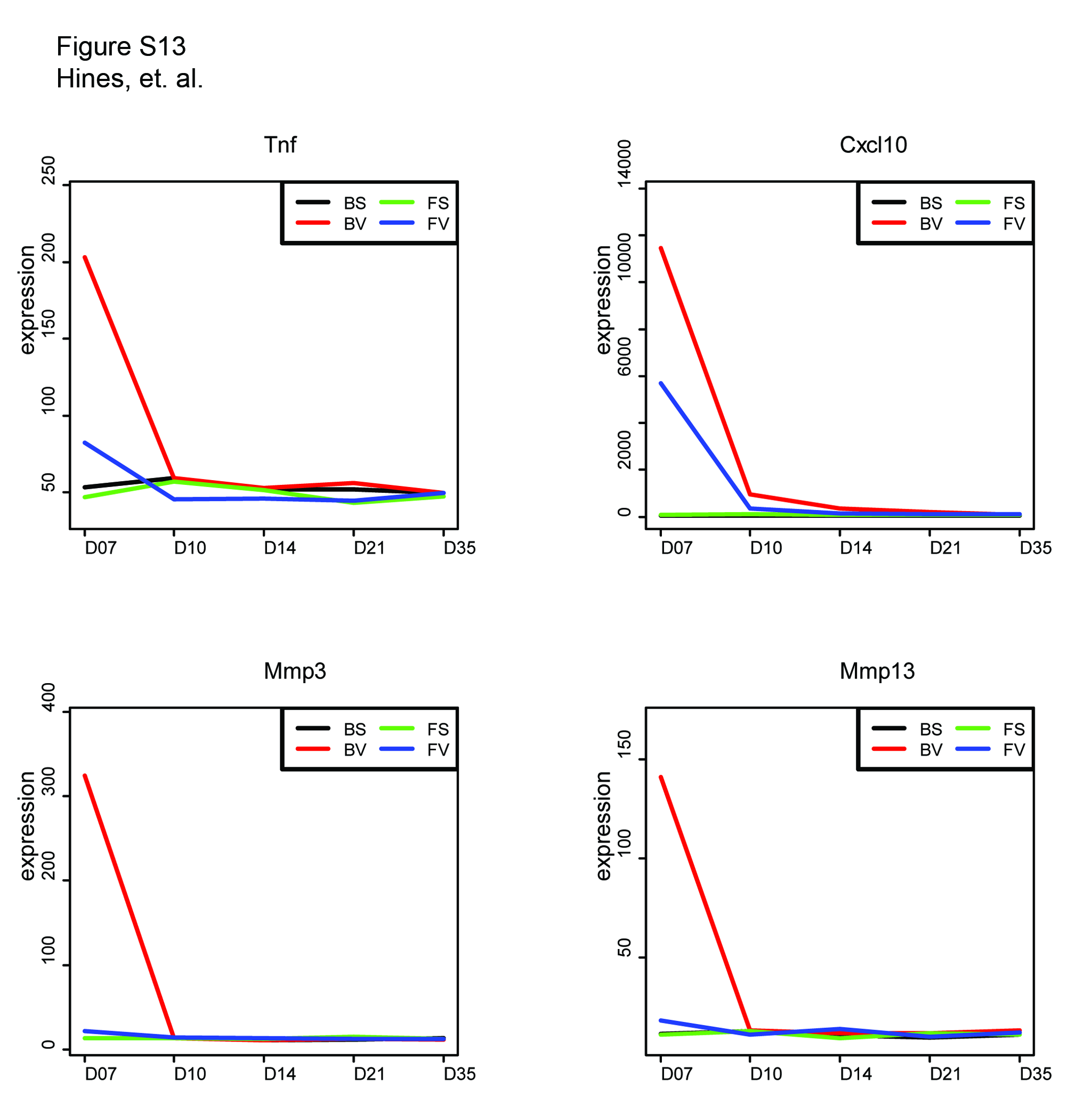

Supplement: Figure S13 — Additional classically activated macrophage effector molecule gene plots. Gene names indicated. F344-sal (FS) (green), BN-sal (BS) (black), F344-virus (FV) (blue), or BN-virus lungs (BV) (red). (TIF) [file pone.0112997.s013.tif]

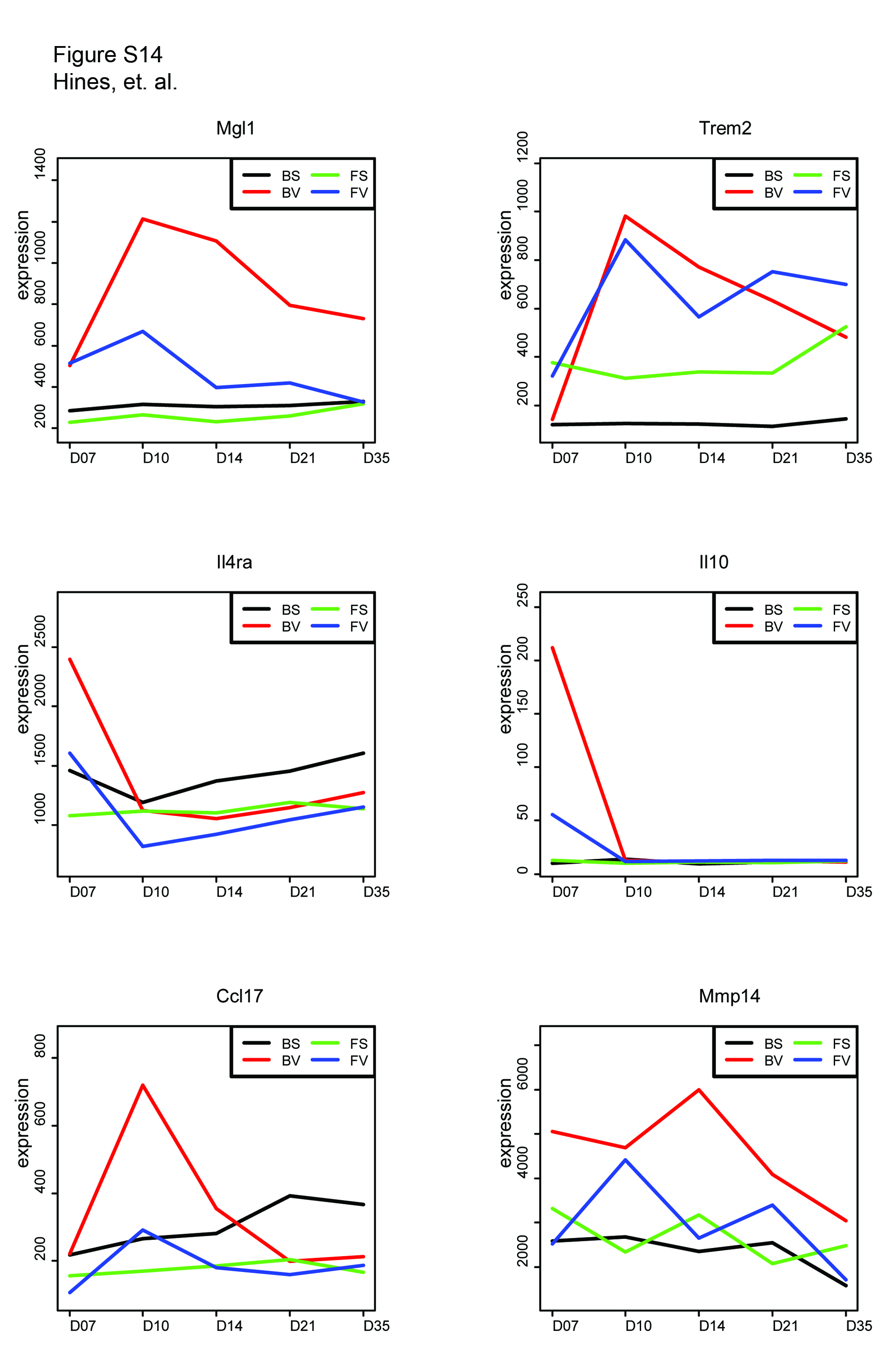

Supplement: Figure S14 — Additional alternatively activated macrophage-related gene plots. Gene names indicated. F344-sal (FS) (green), BN-sal (BS) (black), F344-virus (FV) (blue), or BN-virus lungs (BV) (red). (TIF) [file pone.0112997.s014.tif]

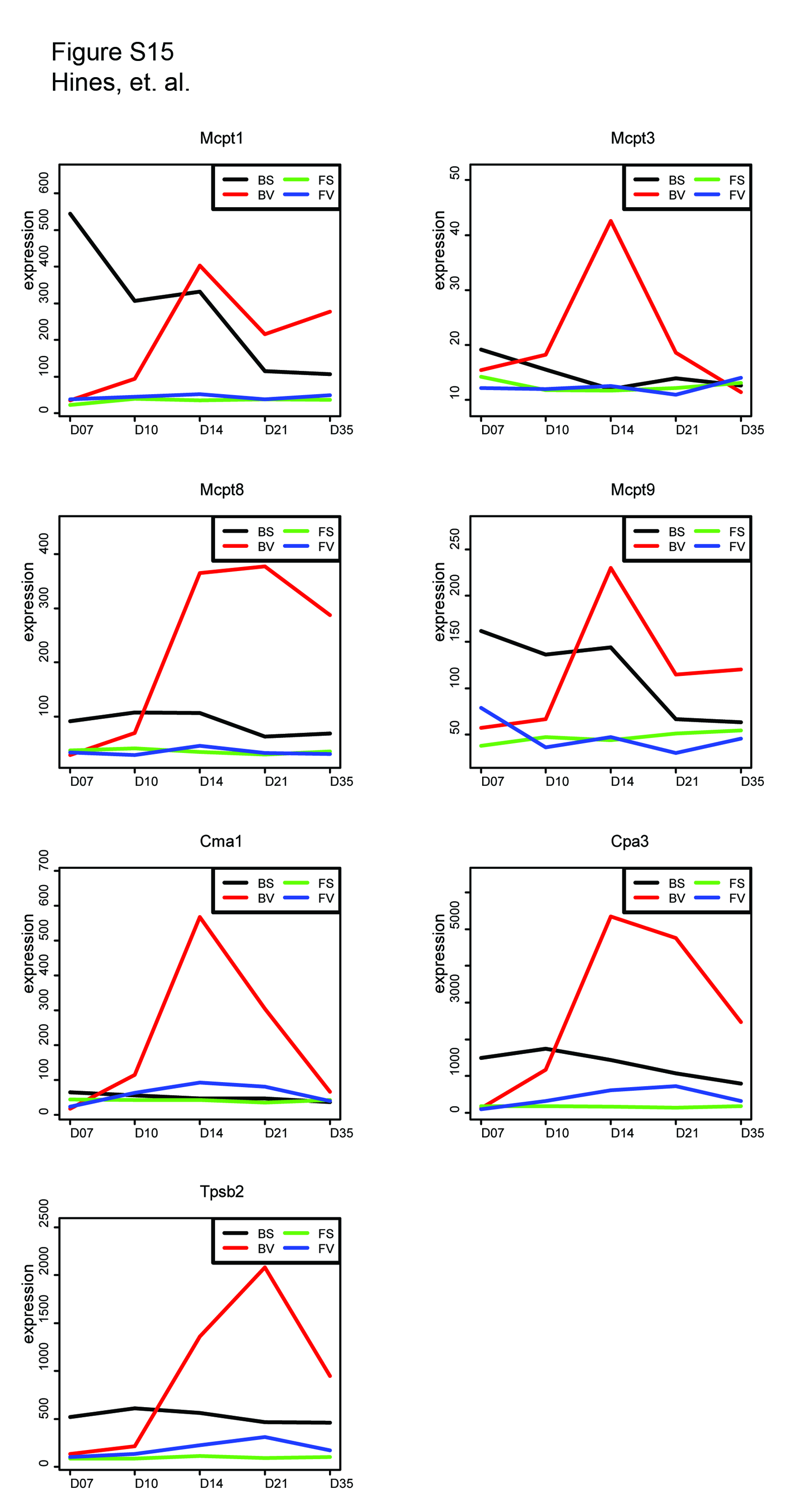

Supplement: Figure S15 — Additional mast cell-related gene plots. Gene names indicated. F344-sal (FS) (green), BN-sal (BS) (black), F344-virus (FV) (blue), or BN-virus lungs (BV) (red). (TIF) [file pone.0112997.s015.tif]

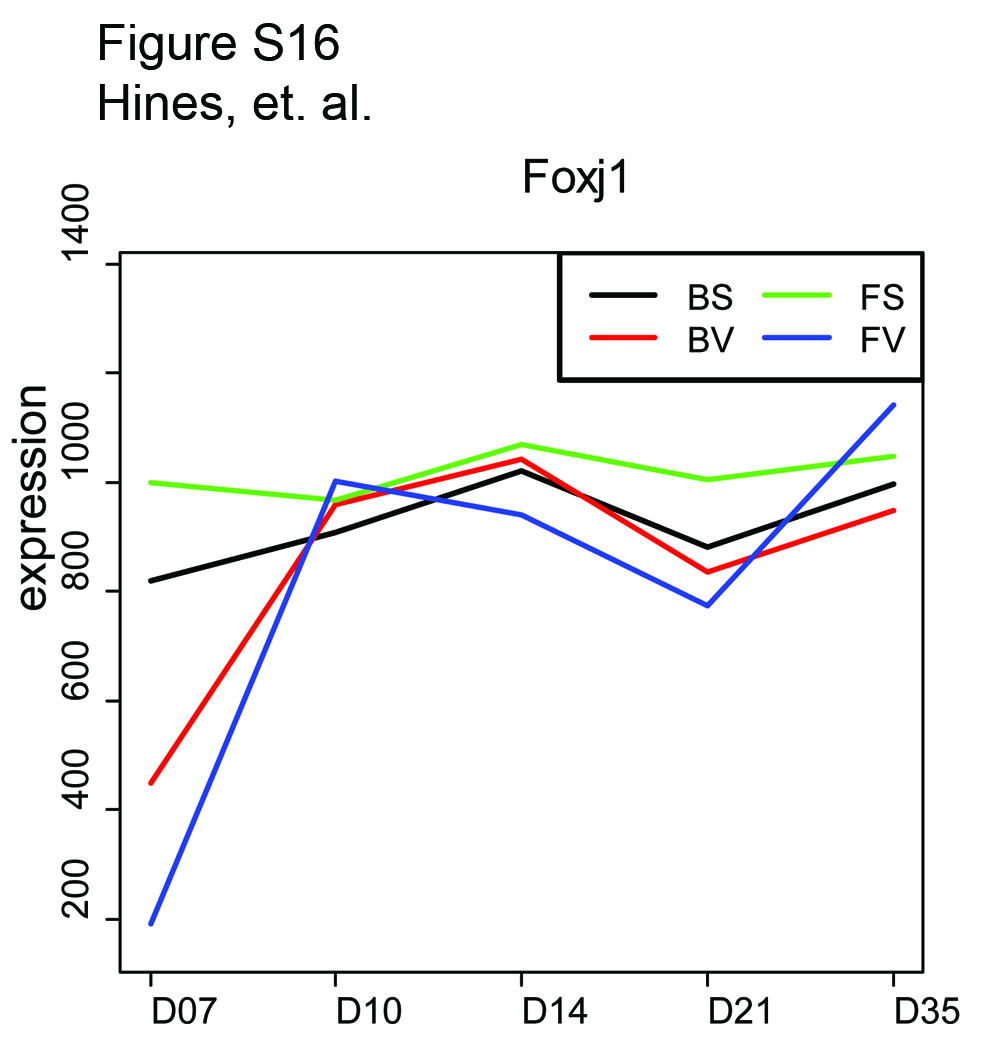

Supplement: Figure S16 — Additional ciliated cell marker gene plot. F344-sal (FS) (green), BN-sal (BS) (black), F344-virus (FV) (blue), or BN-virus lungs (BV) (red). (TIF) [file pone.0112997.s016.tif]
